# Prevalence of Anisakid Nematodes in Fish in China: A Systematic Review and Meta-Analysis

**DOI:** 10.3389/fvets.2022.792346

**Published:** 2022-02-21

**Authors:** Qing Liu, Qi Wang, Jing Jiang, Jun-Yang Ma, Xing-Quan Zhu, Qing-Long Gong

**Affiliations:** ^1^College of Veterinary Medicine, Shanxi Agricultural University, Jinzhong, China; ^2^College of Veterinary Medicine, Jilin Agricultural University, Changchun, China; ^3^College of Life Science, Changchun Sci-Tech University, Changchun, China; ^4^Marine College, Shandong University, Jinan, China; ^5^Key Laboratory of Veterinary Public Health of Higher Education of Yunnan Province, College of Veterinary Medicine, Yunnan Agricultural University, Kunming, China

**Keywords:** anisakid nematodes, fish, prevalence, China, meta-analysis

## Abstract

Anisakidosis, caused by anisakid larvae, is an important fish-borne zoonosis. This study aimed to summarize the prevalence of anisakid infection in fish in China. A systematic review and meta-analysis were performed using five bibliographic databases (PubMed, CNKI, ScienceDirect, WanFang, and VIP Chinese Journal Databases). A total of 40 articles related to anisakid infection in fish in China were finally included. Anisakid nematodes were prevalent in a wide range of fish species, and the overall pooled prevalence of anisakid nematodes in fish in China was 45.5%. Fresh fish had the highest prevalence rate (58.1%). The highest prevalence rate was observed in Eastern China (55.3%), and fish from East China Sea showed the highest prevalence of anisakid nematodes (76.8%). Subgroup analysis by sampling year suggested that the infection rate was higher during the years 2001–2011 (51.0%) than the other periods. Analysis of study quality revealed that the middle-quality studies reported the highest prevalence (59.9%). Compared with other seasons, winter had the highest prevalence (81.8%). The detection rate of anisakid nematodes in muscle was lower (7.8%, 95% CI: 0.0–37.6) than in other fish organs. Our findings suggested that anisakid infection was still common among fish in China. We recommend avoiding eating raw or undercooked fish. Region, site of infection, fish status and quality level were the main risk factors, and a continuous monitoring of anisakid infection in fish in China is needed.

## Introduction

Anisakidosis is a parasitic zoonosis caused by any member of the family Anisakidae, including the genera *Anisakis, Contracaecum*, and *Pseudoterranova* (1–3). The first case of anisakiasis was reported in the Netherlands around 1960, and the total number of anisakiasis cases up to December 2017 was estimated to be about 76,000 throughout the world (4, 5). The pathogenic effects of infection by anisakid nematodes are due mainly to two mechanisms, direct tissue damage and allergic reactions (6). The clinical syndromes can be categorized into gastric anisakiasis, intestinal anisakiasis, ectopic anisakiasis, and allergic anisakiasis (7, 8). Gastric anisakiasis represents about 95% of cases in Japan, and the typical symptom is acute and severe epigastric pain (6, 9). The symptoms of intestinal anisakiasis include intermittent or constant abdominal pain and/or intestinal obstruction, and treatment often requires surgery to remove the worm (7, 10). Moreover, infection with anisakids can lead to life-threatening anaphylaxis (6).

Anisakid nematodes have an indirect life cycle, and crustaceans are intermediate hosts while fish (and mollusks) are paratenic hosts (7, 11, 12). The larvae of anisakid nematodes, especially when located in the musculature, can affect the commercial value of fish (13). Furthermore, anisakid nematodes can lead to disease in fish (13). Humans act only as an accidental host in the life cycle of anisakid nematodes, and the infection can be obtained through consumption of raw or incompletely cooked fish infected with the third-stage larvae of the nematode (14, 15). Hence, infection of fish with anisakid nematodes should be given high priority not only because of anisakiasis in humans, but also because of the economic losses to the fishing industry (13, 16).

Fish are one of the most important food sources in China, and a number of individual studies have reported the prevalence of anisakid nematodes in fish in China. Meanwhile, the first human case of anisakiasis in China has been reported (17). Herein, a systematic review and meta-analysis was performed to analyze the prevalence of anisakid nematodes in fish in China, and the potential related factors were also investigated.

## Materials and Methods

### Search Strategy

This study was performed following the PRISMA guideline (Supplementary Table 1) (18). Five bibliographic databases (VIP Chinese Journal Databases, WanFang, ScienceDirect, CNKI, and PubMed) were used to identify published articles regarding anisakid infection in fishes in China in both Chinese and English up to August 2020. The detailed search strategy and restriction information are recorded in Table 1. Meanwhile, the reference lists of retrieved articles and recent reviews were reviewed. Additionally, we did not contact the original investigators for additional data, and unpublished reports were not retrieved. Endnote X9.3.1 software was utilized to collate information for all studies.

**Table 1:** Detailed search strategy and restrictions.

**Database**	**Limitation**	Search formula^*^
PubMed	All files	(*Anisakis* [MeSH Terms] OR *Anisaki* OR *Pseudoterranova* OR *Contracaccum* OR *Hysterothylacium*) AND (“Fishes” [Mesh] OR fish) AND^*^ (“China”[Mesh] OR People's Republic of China OR Mainland China OR Manchuria OR Sinkiang OR Inner Mongolia)
ScienceDirect	Title, abstract or author-specified keywords: China, fish	Anisakis OR *Hysterothylacium* OR *Pseudoterranova* OR *Contracaccum* AND fish AND China
CNKI	Advanced search and subject term and fuzzy retrieval and synonym extension	“*Anisakis*” (Chinese) and “fish” (Chinese) or “*Hysterothylacium*” (Chinese) and “fish” (Chinese) or “*Pseudoterranova*” (Chinese) and “fish” (Chinese) or “*Contracaccum*” and “fish” (Chinese)
Chongqing VIP	Advanced search and title or keyword and fuzzy retrieval and synonym extension	“*Anisakis*” (Chinese) and “fish” (Chinese) or “*Hysterothylacium*” (Chinese) and “fish” (Chinese) or “*Pseudoterranova*” (Chinese) and “fish” (Chinese) or “*Contracaccum*” and “fish” (Chinese)
WanFang	Advanced search and title or keyword and fuzzy retrieval and synonym extension	“*Anisakis*” (Chinese) and “fish” (Chinese) or “*Hysterothylacium*” (Chinese) and “fish” (Chinese) or “*Pseudoterranova*” (Chinese) and “fish” (Chinese) or “*Contracaccum*” and “fish” (Chinese)

* *“OR” was used to connect the entry terms, and “AND” was used to connect MeSH terms, they are both boolean operators*.

### Study Selection

**Figure 1:**
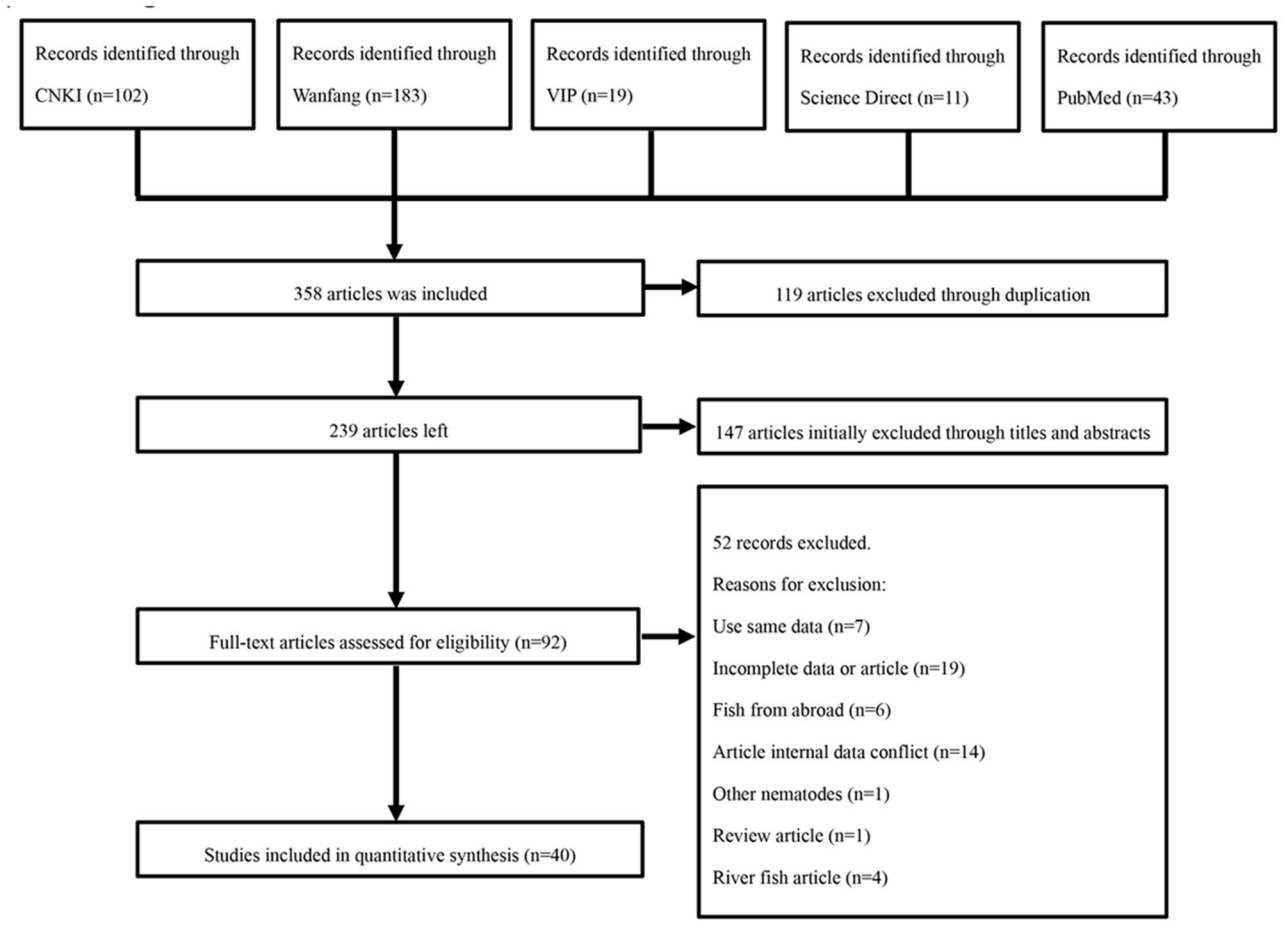
Flow diagram of literature search and selection.

After removing duplicates, the relevant articles were selected through an initial screen of identified abstracts and/or titles and a second screen of full-text articles. Qualified studies needed to meet all of the following criteria: (i) targeted objects must be fish (ii) selected fishing sites within China; (iii) cross-sectional study; (iv) the content of the studies must include the prevalence of anisakid nematodes; and (v) natural infection. Studies with the following characteristics were excluded: using the same data; incomplete data or article; fish from abroad; having internal data conflict; other nematodes; review article; river fish article (Figure 1). Eligibility for inclusion for all studies was evaluated by two independent reviewers (QL and QW). Any disagreements were resolved by the primary reviewer's (QLG) opinion as necessary.

### Data Extraction and Quality Assessment

Two reviewers (QW and JYM) independently extracted the following variables from each included study: Year of sampling, first author, publication year, study region, province, detection method, site of infection, collection season, sea, the total number of fishes, the number of positive samples, fish status, and fish category. The statistical geographic factor data (longitude range, latitude range, annual average rainfall, altitude, annual average temperature, and annual average humidity) were acquired from the National Meteorological Information Center of China Meteorological Administration. The primary reviewer (QLG) confirmed all the extracted data. A “quality” assessment of each included study was made by using criteria derived from the GRADE (Grading of Recommendations Assessment, Development, and Evaluation) approach (19–21). The scoring method was used for grading, and each of the below mentioned criteria was determined as 1 point: (i) randomly sampled; (ii) clear detection method; (iii) provide a detailed description of sampling method; (iv) clear sampling time; and (v) contained four or more risk factors. Studies with total score of four or five points were considered to be of high quality, studies with total scores of 2–3 points were considered to be of moderate quality, whereas studies with lower scores were marked as low quality.

### Statistical Analysis

We performed meta-analysis using the package “meta” (version 4.11-0) in R software (version 3.5.2) (22). Prior to meta-analysis, we tried different methods to fit the data to a Gaussian distribution: double-arcsine transformation (PFT), loga-rithmic conversion (PLN), logit transformation (PLOGIT) and arcsinetransformation (PAS). As indicated by previous studies, PFT has better variance stabilization performance (Table 2) (23–25). The formulas for PFT were as follows:


$$\begin{array}{l} \text{t = arcsin}(\text{sqrt}(\text{r/}(\text{n+1})))\text{ + arcsin}(\text{sqrt}((\text{r+1})\text{/}(\text{n+1})))\\ \text{ se}(\text{t})\text{ = sqrt}(\text{1/}(\text{n+0}.\text{5}))\\ \text{ p = }{\left(\sin\left(\text{t/2}\right)\right)}^{2} \end{array}$$

t, transformed prevalence; *n*, sample size; *r*, positive number; se, standard error.

**Table 2:** Normal distribution test for the normal rate and the different conversion of the normal rate.

**Conversion form**	***W***	***P***
PRAW	0.928	0.013
PLN	NaN	NA
PLOGIT	NaN	NA
PAS	0.954	0.109
PFT	0.941	0.038

*PRAW, original rate; PLN, logarithmic conversion; PLOGIT, logit transformation; PAS, arcsine transformation; PFT, double-arcsine transformation; NaN, meaningless number; NA, missing data*.

Hence, PFT was used for rate conversion in this study. Heterogeneity across all eligible studies was tested by using the Cochran Q-test and *I*-squared statistic. A *P* < 0.05 was considered to indicate statistically significant heterogeneity, and *I*^2^-values of ≥25, ≥50, and ≥75% correspond to low, moderate, and high heterogeneity, respectively (26). Heterogeneity was present, and hence the random effect pooled measure was selected. Forest plots were generated for overall assessment of the results of each included study and the heterogeneity between studies. A funnel plot, trim and fill method and an Egger's test were used to evaluate the publication bias of studies. In addition, the stability of our study was determined by using a sensitivity analysis (27).

Meanwhile, we performed subgroup analysis stratified by the potential risk factors to explore the potential sources of heterogeneity in our meta-analysis (28). The factors included the region (eastern China vs. other regions), the year of collection (2001–2011 vs. other periods), site of infection (others vs. muscle), season (winter vs. spring, summer, and autumn), seas (Bohai Sea vs. East China Sea, South China Sea, and Yellow Sea), fish status (Fresh fish vs. frozen fish, and live fish), and quality level (middle vs. high). In the meta-analysis of prevalence, regional factor is usually the source of heterogeneity. Hence, meta-regression analysis with other risk factors using the provinces as a covariate was conducted to explain the heterogeneity caused by the provinces. The explained heterogeneity of the covariates is expressed in *R*^2^.

Also, potential sources of heterogeneity were explored by subgroup analysis based on geographical factors. We evaluated latitude (30–35° vs. other latitudes), longitude (>120° vs. other longitudes), altitude (>500 m vs. other altitudes), precipitation (1,000–1,500 mm vs. other precipitation categories), humidity (<70% vs. other humidity categories), mean temperature (15–20°C vs. mean temperature of other groups), lowest average temperature (10–15°C vs. lowest average temperature in other groups) and highest average temperature (>25°C vs. highest average temperature in other groups). The R software code for meta-analysis is shown in Supplementary Table 2.

## Results

### Included Studies

In this study, a total of 358 relevant articles were found. Following initial screening and removal of duplicates, 92 articles were identified. Following full text review, 52 articles were further excluded. A further search was carried out based on the reference lists of relevant studies. However, no additional qualified articles were found. Finally, 40 full-text studies published between 2000 and 2020 were included in the quantitative analysis (Figure 1). Of which, eight articles were published in English. According to our quality criteria, 26 publications were of high quality (four or five points), 14 publications showed moderate quality (two or three points), and no publications were of low quality (Table 3, Supplementary Table 3).

**Table 3:** Studies included in the analysis.

**Reference ID**	**Sampling time**	**Province**	Detection methods^*^	**No. tested**	**No. positive**	**Quality score**	**Quality level**
**Eastern China**							
Zhou (29)	1997.11–1998.1	Zhejiang	Morphological identification	172	69	4	High
Ye et al. (30)	2004.04–2005.11	Zhejiang	Morphological identification	281	135	4	High
Zhang et al. (31)	2005.03–2006.03	Shandong	Comprehensive test	123	66	3	Middle
Wang et al. (32)	2007.11–2008.12	Zhejiang	Morphological identification	420	218	4	High
Zhang et al. (33)	2005–2010	Shanghai	Morphological identification	418	55	5	High
Li et al. (34)	2010.01, 05, 06, 09, 11, 12; 2011.01	Shandong	Morphological identification	113	98	5	High
Wen (35)	2011.05	Fujian	Comprehensive test	506	283	4	High
Zhang et al. (36)	2012.04	Jiangsu	Morphological identification	40	32	3	Middle
Liao et al. (37)	2013.11	Shandong	Morphological identification	49	10	4	High
Kong et al. (38)	2011.04–2013.07	Zhejiang	Comprehensive test	122	116	3	Middle
Li et al. (39)	2008.10–2010.10	Zhejiang	Morphological identification	430	269	4	High
Li et al. (40)	2011.04	Shandong	Comprehensive test	85	85	3	Middle
Lin et al. (41)	2012–2016	Fujian	Morphological identification	463	85	5	High
Ye et al. (42)	2016.06–09	Shandong	Morphological identification	169	28	5	High
Zhang et al. (43)	2016.01–12	Shandong	Morphological identification	256	170	4	High
Zhou et al. (44)	2013–2014	Zhejiang	Morphological identification	89	82	4	High
Chen et al. (45)	UN	Zhejiang	Comprehensive test	204	204	3	Middle
Gong et al. (46)	2016.09–2017.06	Shandong	Morphological identification	708	112	5	High
Lu et al. (47)	2015–2017	Shanghai	Morphological identification	633	204	5	High
Xu et al. (48)	2017.03–10	Jiangsu	Comprehensive test	360	128	4	High
Zhang et al. (49)	UN	Zhejiang	Comprehensive test	42	42	2	Middle
Lin et al. (50)	2016.01–2018.12	Fujian	Morphological identification	763	269	5	High
Qiao et al. (51)	2015–2017	Zhejiang	Comprehensive test	140	108	3	Middle
Yang et al. (52)	2016–2017	Fujian	Morphological identification	264	86	4	High
Yang et al. (52)	2016–2017	Jiangsu	Morphological identification	349	154	4	High
Yang et al. (52)	2016–2017	Shandong	Morphological identification	336	85	4	High
Yang et al. (52)	2016–2017	Shanghai	Morphological identification	192	67	4	High
Yang et al. (52)	2016–2017	Zhejiang	Morphological identification	438	155	4	High
Zhang et al. (53)	2018	Jiangsu	Morphological identification	119	78	3	Middle
**Northern China**							
Zhang (54)	2001.10–2002.4.17	Hebei	Morphological identification	607	83	3	Middle
Bi and Zhang (55)	2017	Hebei	UN	246	71	4	High
Ma et al. (56)	2018	Beijing	UN	20	0	3	Middle
Yang et al. (52)	2016–2017	Hebei	Morphological identification	338	43	4	High
**Northeastern China**							
Cai and An (57)	1990–1991	Liaoning	Morphological identification	474	126	4	High
Zhang et al. (58)	UN	Liaoning	Morphological identification	777	221	2	Middle
Bao and Shi (59)	2011.03–09	Liaoning	Morphological identification	413	182	5	High
Du and Zhou (60)	2018.03–10	Liaoning	Morphological identification	193	35	5	High
Geng et al. (61)	2016–2017	Liaoning	Comprehensive test	222	70	4	High
Yang et al. (52)	2016–2017	Liaoning	Morphological identification	321	90	4	High
**South China**							
Sun et al. (62)	1985.3–1985.7	HongKong	Morphological identification	455	249	3	Middle
Liao et al. (63)	1999.05–06	Guangdong	Morphological identification	70	11	3	Middle
Liu et al. (64)	UN	Guangdong	Morphological identification	322	17	2	Middle
Ruan et al. (65)	2004–2008	Guangxi	Morphological identification	86	12	5	High
Huang (66)	2010.04–11	Guangdong	Comprehensive test	410	226	4	High
Chen et al. (67)	2013.02–12	Guangdong	Morphological identification	382	181	5	High
Zhao et al. (68)	2013.12.8–11	Guangdong	Comprehensive test	211	38	4	High
Yang et al. (52)	2016–2017	Guangxi	Morphological identification	184	15	4	High

* *UN, unclear*.

Detection methods*: Comprehensive test: Morphological identification, PCR.

### Pooling and Heterogeneity Analysis

**Figure 2:**
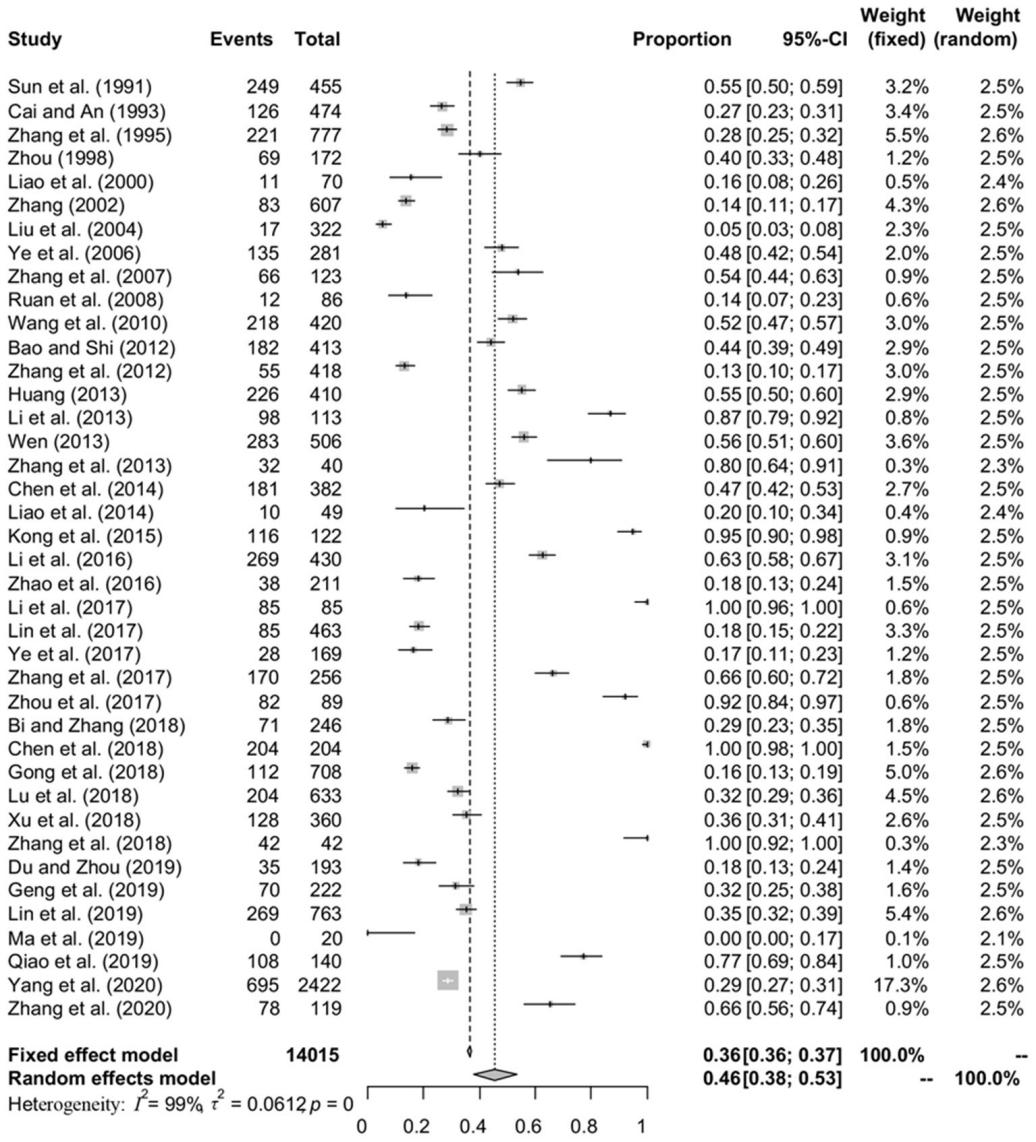
Forest plot of prevalence of anisakids in fish amongst studies conducted in China. The length of the horizontal line represents the 95% confidence interval, and the diamond represents the summarized effect.

A total of 40 studies involving 14,015 fish were included in this meta-analysis. However, high heterogeneity (*I*^2^ = 98.8%, *P* < 0.001) in the selected studies was observed (Table 4, Figure 2). Hence, a random effects model was adopted for the analysis. The overall pooled prevalence of anisakid nematodes in fish in China was 45.5% (95% CI: 37.8–53.3) (Table 4). The included studies covered a variety of fish species, and the prevalence of anisakid nematodes ranged from 0 to 100% (Table 5).

**Table 4:** Pooled prevalence of anisakid nematodes in China.

		**No.** **studies**	**No.** **tested**	**No.** **positive**	% (95% CI^*^)	**Heterogeneity**			**Univariate meta-regression**		
						**χ^2^**	***P*-value**	***I^2^* (%)**	***P*-value**	**Coefficient (95% CI)**	*R^2^* ^*^
Region^*^											15.63%
	Eastern China	29	8,284	3,493	55.3 (45.2–65.2)	2,382.70	0.00	98.8	<0.001	0.330 (0.186–0.474)	
	Northern China	4	1,211	197	13.9 (6.8–22.9)	36.84	<0.01	91.9			
	Northeastern China	6	2,400	724	29.3 (23.3–35.7)	54.49	<0.01	90.8			
	Southern China	8	2,120	749	25.1 (10.9–42.8)	516.20	<0.01	98.6			
**Sampling years**											0.05%
	Before 2001	5	1,814	635	32.9 (21.4–45.5)	118.69	<0.01	97.8			
	2001–2011	12	3,892	1,712	51.0 (36.1–65.8)	977.25	<0.01	98.9	0.040	0.146 (0.007–0.286)	
	After 2011	19	7,485	2,396	37.3 (29.6–45.3)	802.33	<0.01	96.6			
**Site of infection**											0.00%
	Muscle	3	635	58	7.8 (0.0–37.6)	143.79	<0.01	98.6			
	Others	10	2,787	1,285	41.5 (24.0–60.1)	952.84	<0.01	99.0	0.046	0.411 (0.007–0.81.4)	
Season^*^											9.86%
	Autumn	7	1,430	549	60.9 (39.2–80.7)	282.37	<0.01	97.9			
	Spring	7	1,677	829	79.9 (58.2–95.2)	412.66	<0.01	98.5			
	Summer	3	757	222	78.0 (16.2–100.0)	102.75	<0.01	98.1			
	Winter	4	303	126	81.8 (23.7–100.0)	221.81	<0.01	98.6	0.166	−0.198 (−0.479–0.082)	
Sea^*^											11.21%
	Bohai sea	2	1,020	265	27.5 (4.4–60.6)	118.12	<0.01	99.2	0.084	−0.395 (−0.842–0.053)	
	East China sea	8	2,402	1,361	76.8 (56.5–92.1)	747.42	<0.01	99.1			
	South China sea	3	707	276	27.8 (5.8–58.0)	117.49	<0.01	98.3			
	Yellow sea	4	370	259	71.4 (32.5–97.6)	174.82	<0.01	98.3			
**Fish status**											28.90%
	Fresh fish	16	5,973	2,435	58.1 (43.6–72.0)	1,769.92	0.00	99.2	0.003	0.383 (0.130–0.636)	
	Frozen fish	2	205	28	5.9 (0.0–30.9)	13.83	<0.01	92.8			
	Live fish	5	1,530	503	29.2 (12.5–49.4)	242.38	<0.01	98.3			
**Quality level**											8.00%
	High	26	10,889	3,851	38.0 (31.4–44.9)	1,913.33	<0.01	99.3			
	Middle	14	3,126	1,312	59.9 (37.6–80.2)	1,302.76	0.00	98.1	0.009	0.219 (0.054–0.385)	
Total		40	14,015	5,163	45.5 (37.8–53.3)	3,282.18	0.00	98.8			

*CI*

* *, Confidence interval*.

Region*: *: Eastern China: Fujian, Jiangsu, Shandong, Shanghai, Zhejiang; Northern China: Beijing, Hebei; Northeastern China: Liaoning; Southern China: Guangdong, Guangxi, Hainan*.

*R^2^, Proportion of between-study variance explained by joint test with provinces as a covariate*.

Part*: Other: Body cavity, gonad, various tissues, and organs.

Season*: *: Spring: March–May; Summer: June–August; Autumn: September–November; Winter: December–January*.

**Table 5:** Estimated pooled prevalence in different species of fish.

**Fish category**	**No. studies**	**No. tested**	**No. positive**	**% Prevalence**	**% (95% CI)**
*Ablennes hians*	1	43	0	0.0	0.0–4.0
*Abudefduf septemfasciatus*	2	16	0	0.0	0.0–11.6
*Acanthocepola limbata*	2	34	19	56.1	38.2–73.3
*Acanthogobius flavimanus*	1	21	18	85.7	66.9–98.0
*Acanthopagrus australis*	1	4	0	0.0	0.0–38.9
*Acanthopagrus latus*	5	63	10	11.2	2.2–23.8
*Acanthopagrus schlegelii*	4	66	21	35.2	2.0–79.1
*Aciusthalassiaus*	1	17	11	64.7	40.2–86.0
*Albiflora croaker*	1	31	6	19.4	7.1–35.4
*Alectis ciliaris*	1	1	1	100.0	0.0–100.0
*Alepes melanopterus*	1	2	1	50.0	0.0–100.0
*Anguilla japonica*	1	1	0	0.0	0.0–100.0
*Anguillidae*	3	32	8	23.3	8.6–41.4
*Anoplopoma fimbria*	2	19	2	7.0	0.0–36.2
*Apogon carinatus*	1	3	2	66.7	5.9–100.0
*Apogon ellioti*	1	2	1	50.0	0.0–100.0
*Apogon semilineatus*	1	6	1	16.7	0.0–58.6
*Apteronotus albifrons*	1	7	0	0.0	0.0–23.2
*Argyrosomus argentatus*	1	8	1	12.5	0.0–46.2
*Argyrosomus macrocephalus*	1	3	3	100.0	50.0–100.0
*Aristichthys nobilis*	3	50	3	4.9	0.0–22.0
*Astroconger myriaster*	2	67	25	37.5	26.0–49.4
*Atule mate*	1	3	2	66.7	5.9–100.0
*Bembras japonicus*	1	7	3	42.9	8.1–81.4
*Blotchy rock cod*	1	1	0	0.0	0.0–100.0
*Branchiostegus albus*	1	4	1	25.0	0.0–79.3
*Branchiostegus argentatus*	4	26	10	40.5	5.0–81.5
*Branchiostegus japonicus*	1	9	0	0.0	0.0–18.3
*Branchiostegus wardi*	1	5	3	60.0	13.8–98.2
*Brotula barbata*	1	10	1	10.0	0.0–38.1
*Calliurichthysjaponicus*	1	1	1	100.0	0.0–100.0
*Caranx malabaricus*	1	2	2	100.0	30.3–100.0
*Carassius auratus*	1	73	28	38.4	27.5–49.8
*Centroberyx lineatus*	1	2	2	100.0	30.3–100.0
*Chaetodontidae butterflyfish*	1	7	2	28.6	1.0–68.2
*Channa argus*	1	1	0	0.0	0.0–100.0
*Chelidonichthys kumu*	3	14	7	54.4	18.4–88.5
*Choerodon azurio*	1	4	2	50.0	3.0–97.1
*Chorinemus moadetta*	1	5	1	20.0	0.0–67.5
*Cirrhinus molitorella*	2	22	2	16.3	0.0–96.7
*Claris fuscus Lacepede*	1	3	1	33.3	0.0–94.1
*Cleisthenes herzensteini*	2	24	12	50.0	28.3–71.6
*Cleisthenes pinetorum*	1	1	0	0.0	0.0–100.0
*Clupanodon punctatus*	1	8	6	75.0	38.5–99.2
*Clupea pallasi*	3	22	9	45.4	0.0–100.0
*Cociella crocodilus*	2	5	4	86.2	21.3–100.0
*Coilia ectenes*	2	26	8	30.7	13.7–50.6
*Coilia mystus*	2	88	3	5.9	0.0–34.3
*Collichthys lucidus*	2	16	3	15.4	0.0–48.4
*Collichthys niveatus*	5	125	67	53.7	44.6–62.6
*Cololabis saira*	4	75	22	25.6	4.3–54.8
*Conger myriaster*	1	204	204	100.0	99.2–100.0
*Cynoglossus joyneri*	1	14	0	0.0	0.0–11.9
*Cynoglossus robustus*	8	101	20	2.3	0.0–23.0
*Cynoglossus semilaevis*	1	9	0	0.0	0.0–18.3
*Dasyatis akajei*	1	4	0	0.0	0.0–38.9
*Decapterus maruadsi*	7	122	55	57.8	12.2–95.1
*Dentex tumifrons*	5	29	7	33.1	0.0–84.6
*Ditrema temmincki*	5	257	79	11.0	0.0–39.3
*Echeneis naucrates*	1	3	3	100.0	50.0–100.0
*Enedrias fangi wang&wang*	1	2	1	50.0	0.0–100.0
*Engraulis japonicus*	2	192	29	11.5	3.6–21.9
*Epinehelus moara*	4	21	4	37.4	0.0–100.0
*Epinephelus*	3	19	4	10.5	0.0–51.2
*Epinephelus amblycephalus*	1	3	3	100.0	50.0–100.0
*Epinephelus areolatus*	2	5	3	60.3	10.3–99.7
*Epinephelus awoara*	4	13	3	32.7	0.0–99.0
*Epinephelus chlorostigma*	1	1	1	100.0	0.0–100.0
*Epinephelus epistictus*	1	1	1	100.0	0.0–100.0
*Epinephelus fasciatus*	1	3	2	66.7	5.9–100.0
*Epinephelussp*	1	42	5	11.9	3.6–23.7
*Eupleurogrammus muticus*	1	2	2	100.0	30.3–100.0
*Formio niger*	3	10	2	15.2	0.0–50.2
*Fuscous spinefoot*	1	1	0	0.0	0.0–100.0
*Gadus*	1	12	0	0.0	0.0–13.9
*Gadus morhua*	3	33	26	75.4	5.1–100
*Germs acinaces*	1	5	3	60.0	13.8–98.2
*Gerreomorpha jaρonica*	1	1	1	100.0	0.0–100.0
*Girella punctata*	2	40	8	19.4	7.5–34.6
*Gymnocorymbus ternetzi*	1	24	20	83.3	65.4–96.0
*Harengula zunasi*	2	34	5	41.8%	0.0–100.0
*Harpadon nehereus*	8	152	52	40.2	14.6–68.6
*Hemirhamphus sajori*	1	36	29	80.6	65.8–92.1
*Hemisalanx prognathus*	2	17	0	0.0	0.0–1.8
*Hexagrammos otakii*	1	125	39	31.2	23.4–39.6
*Hoplobrotula armata*	1	1	1	100.0	0.0–100.0
*Hypomesus olidus*	2	83	2	1.8	0.0–6.5
*Ilisha elongata*	10	75	15	16.0	6.6–27.5
*Inimicus japonicus*	1	2	0	0.0	0.0–69.7
*Japanese Spanish mackerel*	1	2	0	0.0	0.0%−69.7
*Johnius belengerii*	1	12	10	83.3	56.1–99.6
*Johnius grypotus*	2	11	2	35.9	0.0–100.0
*Kaiwarinus equula*	1	3	2	66.7	5.9–100.0
*Katsuwonus pelamis*	1	2	0	0.0	0.0–69.7
*Konosirus punctatus*	1	75	13	17.3	9.5–26.8
*Larimichthys*	1	34	1	2.9	0.0–12.2
*Larimichthys crocea*	13	556	49	11.3	1.6–25.9
*Larimichthys polyactis*	21	1,492	705	58.0	42.7–72.5
*Lateolabrax japonicus*	11	118	26	17.4	0.3–45.2
*Lepidotrigla microptera*	3	28	16	64.1	26.7–93.5
*Lepidotrigla micropterus*	1	4	4	100.0	61.2–100.0
*Lepturacanthus savala*	1	8	3	37.5	6.7–74.1
*Lophiiformes*	1	20	0	0.0	0.0–8.4
*Lophius litulon*	7	82	79	99.5	91.5–100.0
*Lutjanus argentimaculatus*	2	13	0	0.0	0.0–10.3
*Lutjanus erythropterus*	3	14	13	17.7	0.2–46.8
*Lutjanus fulviflamma*	1	5	0	0.0	0.0–31.7
*Lutjanus fulvus*	1	6	5	83.3	41.4–100.0
*Lutjanus lutjanus*	1	9	9	100.0	81.7–100.0
*Lutjanus ophuysenii*	1	7	6	85.7	48.3–100.0
*Lutjanus russellii*	1	7	1	14.3	0.0–51.7
*Megalaspis cordyla*	3	19	2	5.1	0.0–20.2
*Mene maculata*	3	18	14	87.8	32.8–100.0
*Miichthys miiuy*	10	105	36	37.5	17.8–59.1
*Monopterus albus*	1	1	0	0.0	0.0–100.0
*Mugil cephalus*	3	19	5	19.3	0.0–92.2
*Mullidae subvittatus*	1	6	6	100.0	73.2–100.0
*Muraenesox cinereus*	10	152	120	76.4	51.5–91.3
*Mustelusmanazo*	1	5	0	0.0	0.0–31.7
*Navodon modestus*	1	4	2	50.0	3.0–97.1
*Nemipterus bathybius*	1	12	12	100.0	86.1–100.0
*Nemipterus japonicus*	1	14	10	100.0	83.5–100.0
*Nemipterus virgatus*	6	68	30	37.5	0.0–96.1
*Neτnipterus tolu*	1	1	1	100.0	0.0–100.0
*Nibea albiflora*	8	115	25	27.6	4.4–57.7
*Oncorhynchus*	4	101	0	0.0	0.0–2.1
*Oncorhynchus keta*	1	25	0	0.0	0.0–6.8
*Oncorhynchus mykiss*	1	2	0	0.0	0.0–69.7
*Ophiocephalus argus*	1	20	20	100.0	91.6–100.0
*Oreochromis*	2	2	0	0.0	0.0–78.7
*Pagrosomus major*	67	73	46	67.9	30.1–70.0
*Pagrus major*	1	1	0	0.0	0.0–100.0
*Pampus argenteus*	9	124	9	4.4	0.0–15.8
*Pangsius suthi*	1	4	0	0.0	0.0–38.9
*Paralichthys lethostigma*	2	17	5	28.7	7.9–54.3
*Paralichthys olivaceus*	7	183	29	21.7	3.7–45.9
*Parapercis cylindrica*	1	10	2	20.0	0.5–51.3
*Parapristipoma trilineatum*	1	11	0	0.0	0.0–15.1
*Parargyrops edita*	1	17	14	82.4	60.0–97.4
*Parastromateus niger*	1	1	0	0.0	0.0–100.0
*Parupeneus chrysopleuron*	1	9	4	44.4	13.0–78.1
*Pelates quadrilineatus*	1	32	16	50.0	32.6–67.4
*Pennahia argentata*	9	119	68	56.6	24.6–86.2
*Pentapus setosus*	1	3	1	33.3	0.0–94.1
*Perca fluviatilis*	5	135	3	0.0	0.0–1.7
*Perea flavescens*	1	3	2	66.7	5.9–100.0
*Periophthalmus cantonensis*	1	16	0	0.0	0.0–10.5
*Platichthys bicoloratus*	1	16	7	43.8	20.1–68.9
*Platycephalus indicus*	3	54	28	39.1	0.0–97.7
*Plectorhinchus cinctus*	4	32	5	13.5	2.2–29.5
*Plectorhinchus nigrus*	1	6	0	0.0	0.0–26.8
*Plectorhynchispictus*	1	6	5	83.3	41.4–100.0
*Plectorhynchus cinctus*	3	78	26	28.8	16.3–42.7
*Pleuronectiformes*	1	59	7	11.9	4.7–21.5
*Pleuronichthys cornutus*	1	10	0	0.0	0.0–16.5
*Pneumatophorus japonicus*	24	583	482	75.8	61.0–88.3
*Pogonoperca punctata*	1	12	3	25.0	3.9–53.9
*Pomfret*	1	155	1	0.7	0.0–2.8
*Priacanthus boops*	1	1	1	100.0	0.0–100.0
*Priacanthus cruentatus*	2	7	5	77.3	27.9–100.0
*Priacanthus macracanthus*	2	9	3	27.9	0.0–100.0
*Priacanthus tayenus*	5	24	16	70.4	9.3–100.0
*Pristigenys niphonia*	1	4	3	75.0	20.8–100.0
*Pristipomoides typus*	1	7	5	71.4	31.8–99.0
*Prognichthys agoo*	1	10	7	70.0	37.5–95.0
*Psenopsis anomala*	2	19	2	7.5	0.0–44.8
*Pseudopriacanthus niphonius*	1	5	3	60.0	13.8–98.2
*Pseudorhombus arsius*	1	1	1	100.0	0.0–100.0
*Pseudorhombus cinnamoneus*	1	85	85	100.0	98.0–100.0
*Pseudosciaena polyactis*	2	20	20	95.1	72.1–100.0
*Rachycentron canadum*	2	4	2	50.0	0.0–100.0
*Raja hollandi*	1	5	0	0.0	0.0–31.7
*Raja porosa*	3	32	7	15.4	0.0–62.7
*Rastrelliger kanagurta*	2	15	10	76.3	5.8–100.0
*Rock fish*	1	8	0	0.0	0.0–20.4
*Sardine*	4	72	2	0.9	0.0–9.7
*Saurida elongata*	2	36	28	78.2	62.6–90.9
*Saurida filamentosa*	1	2	2	100.0	30.3–100.0
*Scatophagus argus*	3	21	1	1.0	0.0–14.8
*Sciaenidae*	2	60	9	14.9	6.6–25.4
*Sciaenops ocellatus*	1	18	16	88.9	69.4–99.8
*Scolopsis taeniopterus*	1	1	1	100.0	0.0–100.0
*Scolopsis trilineata*	1	4	1	25.0	0.0–79.3
*Scolopsis vosmeri*	1	9	1	11.1	0.0–41.8
*Scomber australasicus*	1	4	4	100.0	61.2–100.0
*Scomber japonicus*	1	20	13	65.0	42.5–84.7
*Scomberomorus commerson*	1	10	2	20.0	0.5–51.3
*Scomberomorus guttatus*	1	4	1	25.0	0.0–79.3
*Scomberomorus niphonius*	19	468	214	36.9	22.9–51.9
*Scophthalmus maximus*	6	101	2	0.0	0.0–0.0
*Sea catfish*	1	4	0	0.0	0.0–38.9
*Sebastiscus marmoratus*	3	88	24	27.1	6.9–52.9
*Sebastodes fuscescens*	2	22	19	96.0	74.0–100.0
*Secutor insidiator*	1	2	1	50.0	0.0–100.0
*Secutor ruconius*	1	3	1	33.3	0.0–94.1
*Selaroides leptolepis*	1	2	2	100.0	30.3–100.0
*Seriola lalandi*	1	4	0	0.0	0.0–38.9
*Setipinna tenuifilis*	3	104	20	22.5	0.0–71.1
*Siganus argenteus*	1	3	0	0.0	0.0–50.0
*Siganus fuscescens*	3	47	4	3.5	0.0–14.1
*Sillagojaponica*	1	5	2	40.0	1.9–86.2
*Soleidae*	1	12	0	0.0	0.0–13.9
*Sphyraena pingais*	2	5	3	70.0	1.4–100.0
*Sphyraena pinguis*	1	5	0	0.0	0.0–31.7
*Sphyraenus*	3	53	26	47.8	0.0–100.0
*Stingray*	1	1	0	0.0	0.0–100.0
*Stromateoides argenteus*	1	36	0	0.0	0.0–4.7
*Stromateus*	1	3	2	66.7	5.9–100.0
*Synanceia verrucosa*	1	4	0	0.0	0.0–38.9
*Taius tumifrons*	1	24	21	87.5	70.7–98.3
*Talismania longifilis*	1	4	0	0.0	0.0–38.9
*Tenualosa reevesii*	4	24	0	0.0	0.0–4.5
*Terapon jarbua*	1	11	2	18.2	0.5–47.4
*Thamnaconus modestus*	4	31	3	6.7	0.0–21.1
*Thamnaconus septentrionalis*	1	6	0	0.0	0.0–26.8
*Therapon oxyrhynchus*	1	25	3	12.0	1.7–28.2
*Therapon theraps*	2	29	2	8.1	0.0–27.6
*Thunnus alalunga*	4	36	6	7.5	0.0–38.1
*Trachinocephalus myops*	1	7	7	100.0	76.8–100.0
*Trachinotus blochii*	1	2	1	50.0	0.0–100.0
*Trachinotus ovatus*	13	148	1	0.0	0.0–1.0
*Trachurus japonicus*	7	108	85	81.0	55.8–98.3
*Triaenopogon barbatus*	1	1	1	100.0	0.0–100.0
*Trichiurus haumela*	2	109	103	94.7	89.4–98.4
*Trichiurus lepturus*	25	1,631	840	69.8	57.1–87.3
*Tridentiger trigonoephalus*	1	20	2	10.0	0.2–27.8
*Trisotropis dermopterus*	1	1	1	100.0	0.0–100.0
*Tuna Rubrum*	1	5	0	0.0	0.0–31.7
*Tylosurus anastomella*	1	4	1	25.0	0.0–79.3
*Tylosurus melanotus*	1	21	8	38.1	18.3–60.0
*Upeneus luzonius*	1	2	1	50.0	0.0–100.0
*Upeneus moluccensis*	1	2	2	100.0	30.3–100.0
*Upeneus sulphureus*	2	14	8	60.3	24.7–91.8
*Uranoscopus japonicus*	1	7	6	85.7	48.3–100.0
*Zebrias zebra*	1	1	0	0.0	0.0–100.0
*Zoarces slongatus*	1	2	0	0.0	0.0–69.7
*Zoarcidae*	1	22	2	9.1	0.2–25.5
*Zuta jifish*	1	23	3	13.0	1.8–30.5

**Figure 3:**
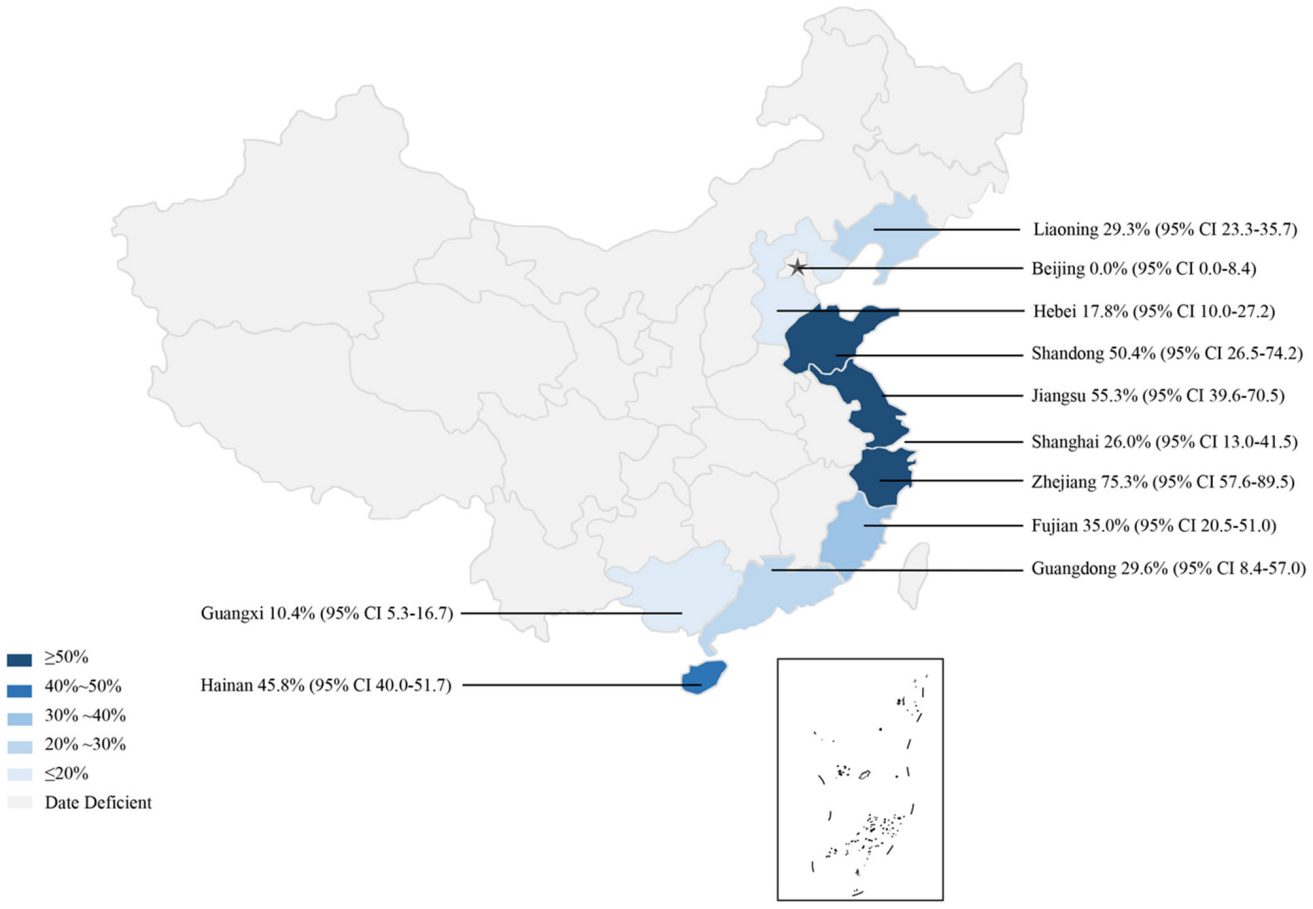
Map of anisakid infection in fish amongst studies conducted in China.

In the subgroup analysis, a random effect model was selected due to the fact that significant heterogeneity was observed (Table 4). The subgroup analysis based on geographical areas suggested that eastern China had the highest prevalence rate (55.3%, 95% CI: 45.2–65.2), and fish in East China Sea showed the highest point estimate of prevalence of anisakid nematodes (76.8%, 95% CI: 56.5–92.1). At the single province level, Zhejiang Province had the highest rate of 75.3% (1,398/2,338; 95% CI: 57.6–89.5) (Table 6). No anisakid nematodes were found in fish in Beijing City (Table 6, Figure 3).

**Table 6:** Estimated pooled prevalence of anisakid nematodes by provinces in China.

**Province**	**No. studies**	**Region**	**No. tested**	**No. positive**	**% Prevalence**	**% (95% CI)**
Beijing	1	Northern China	20	0	0.0	0.0–8.4
Fujian	4	Eastern China	1,996	723	35.0	20.5–51.0
Guangdong	5	Southern China	1,120	347	29.6	8.4–57.0
Guangxi	2	Southern China	270	27	10.4	5.3–16.7
Hainan	1	Southern China	275	126	45.8	40.0–51.7
Hebei	3	Northern China	1,191	197	17.8	10.0–27.2
Jiangsu	4	Eastern China	868	392	55.3	39.6–70.5
Liaoning	6	Northeastern China	2,400	724	29.3	23.3–35.7
Shandong	8	Eastern China	1,839	654	50.4	26.5–74.2
Shanghai	3	Eastern China	1,243	326	26.0	13.0–41.5
Zhejiang	10	Eastern China	2,338	1,398	75.3	57.6–89.5
Total	47		13,560	4,914	42.7	35.5–50.1

The subgroup analysis by sampling years demonstrated that the infection rate was higher during 2000–2011 (51.0%, 95% CI: 36.1–65.8) than other periods. Compared with other seasons, autumn had the lowest prevalence rate (60.9%, 95% CI: 39.2–80.7) (Table 4).

Analysis of study quality indicated that the middle-quality studies reported the highest prevalence rate (59.9%, 95% CI: 37.6–80.2). The detection rate of anisakid nematodes in muscle was lower (7.8%, 95% CI: 0.0–37.6) than in other fish organs. The meta-regression analysis showed that the heterogeneity can be explained by the province ranges from 0.00 to 31.93% after joint analysis with province (Table 4).

We also evaluated the impact of geographical and climatic parameters on prevalence and calculated the latitude range (30–35°; 68.6%, 95% CI: 51.9–83.1), the longitude range (>120°; 61.4%, 95% CI: 47.8–74.2), and altitude (<100; 54.1%, 95% CI: 42.5–65.5). Compared with other groups, the prevalence of anisakid nematodes in fish in these geographic ranges was significantly higher (*P* < 0.05), which may account for the heterogeneity (Table 7).

**Table 7:** Pooled prevalence of geographical factors.

		**No.** **studies**	**No.** **tested**	**No.** **positive**	% (95% CI*)	**Heterogeneity**			**Univariate meta-regression**		
						**χ^2^**	***P*-value**	***I^2^* (%)**	***P*-value**	**Coefficient (95% CI)**	***R^**2**^***
**North latitude**											0.00%
	30 less	7	1,024	368	27.6 (12.3–46.1)	186.47	<0.01	96.8			
	30–35	11	2,790	1,461	68.6 (51.9–83.1)	785.44	<0.01	98.7	0.001	0.344 (0.148–0.54.1)	
	35 more	13	3,759	1,161	37.9 (24.4–52.3)	909.56	<0.01	98.7			
**East longitude**											0.00%
	110 less	3	86	12	26.5 (0.0–87.9)	66.87	<0.01	97.0			
	110–120	19	2,220	648	24.2 (14.9–34.9)	222.26	<0.01	96.4			
	120 more	19	5,267	2,330	61.4 (47.8–74.2)	1,759.66	0.00	99.0	0.000	0.387 (0.184–0.590)	
**Altitude (0.1 m)**											0.00%
	100 less	11	2,617	1,132	54.1 (42.5–65.5)	341.52	<0.01	97.1			
	100–500	13	2,672	1,192	51.8 (30.5–72.9)	1,391.70	<0.01	99.1			
	500 more	7	1,853	666	31.7 (17.3–48.1)	273.50	<0.01	97.8	0.075	−0.218 (−0.458–0.022)	
**Average rainfall (mm)**											0.00%
	1,000 less	12	3,169	1,065	47.9 (31.7–64.2)	911.73	<0.01	98.8			
	1,000–1,500	7	1,467	774	40.1 (23.8–57.7)	246.71	<0.01	97.6	0.492	0.070 (−0.129–0.268)	
	1,500 more	6	1,582	571	39.2 (26.7–52.4)	113.34	<0.01	95.6			
**Average humidity (%)**											0.00%
	70 less	9	2,725	779	30.3 (16.3–46.4)	567.47	<0.01	98.6	0.067	−0.177 (−0.368–0.013)	
	70–80	14	3,209	1,459	47.4 (35.0–60.0)	637.65	<0.01	98.0			
	80 more	5	616	285	48.5 (27.8–69.5)	90.46	<0.01	95.6			
**Average temperature (** **°** **C)**											0.00%
	15 less	12	2,982	940	38.6 (22.9–55.7)	908.08	<0.01	98.8			
	15–20	9	2,544	1,215	56.6 (45.1–67.8)	259.81	<0.01	96.9	0.024	0.223 (0.028–0.417)	
	20 more	7	1,024	368	27.6 (12.3–46.1)	186.47	<0.01	96.8			
**Maximum temperature (** **°** **C)**											0.00%
	20 less	13	2,445	727	42.3 (26.8–58.6)	951.43	<0.01	98.7			
	20–25	8	2,390	1,126	53.1 (40.6–65.4)	256.78	<0.01	97.3			
	25 more	7	1,024	368	27.6 (12.3–46.1)	186.47	<0.01	96.8	0.094	−0.191 (−0.415 to 0.032)	
**Lowest temperature (** **°** **C)**											0.00%
	10 less	8	1,687	522	37.2 (16.8–60.3)	494.61	<0.01	98.8			
	10–15	14	3,206	1,429	52.7 (38.6–66.5)	747.62	<0.01	98.4	0.035	0.201 (0.014–0.389)	
	15 more	8	1,657	572	27.7 (15.9–41.2)	187.32	<0.01	98.3			

### Publication Bias and Sensitivity Analysis

**Figure 4:**
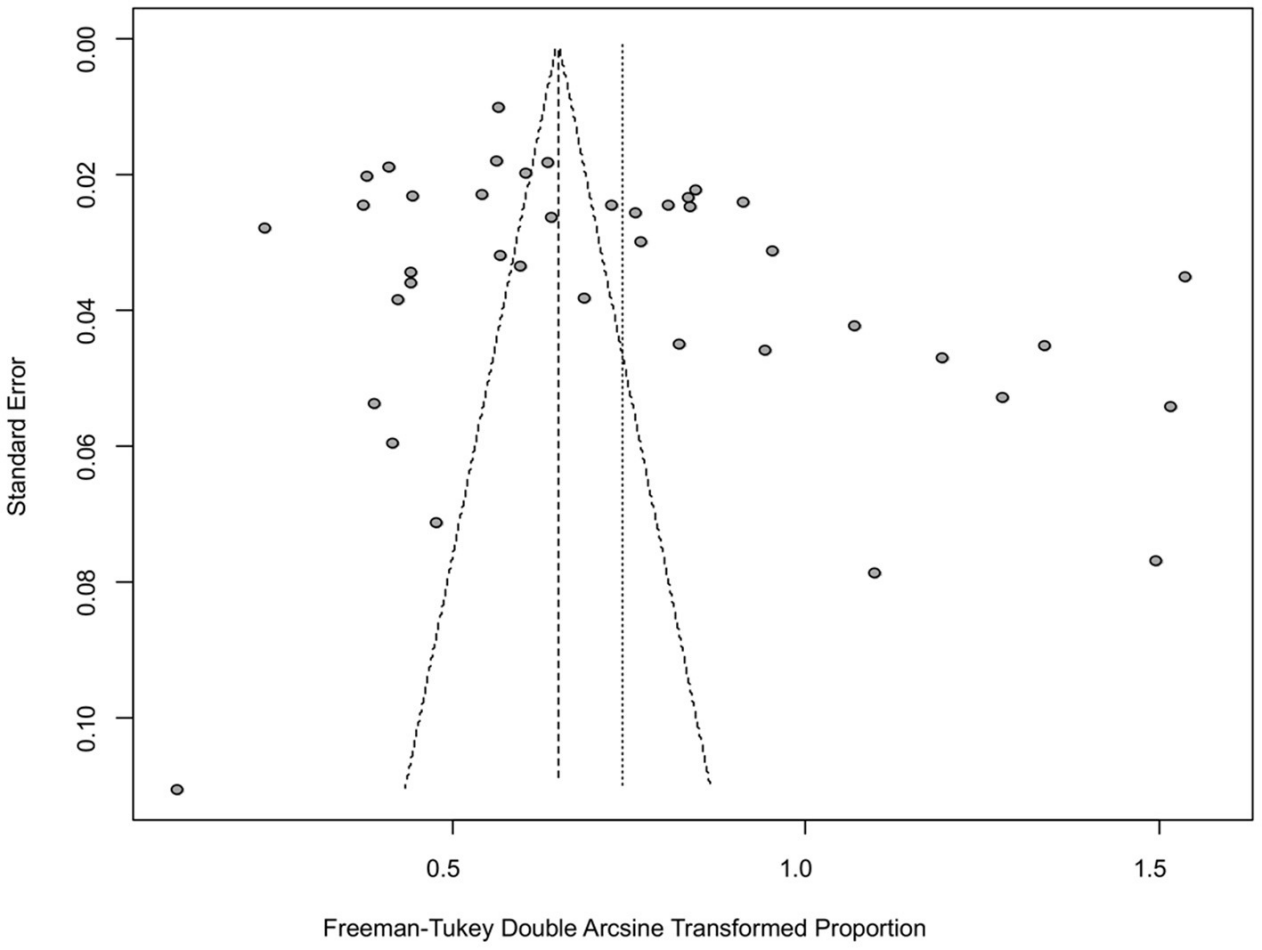
Funnel plot with pseudo 95% confidence interval limits for the examination of publication bias.

**Figure 5:**
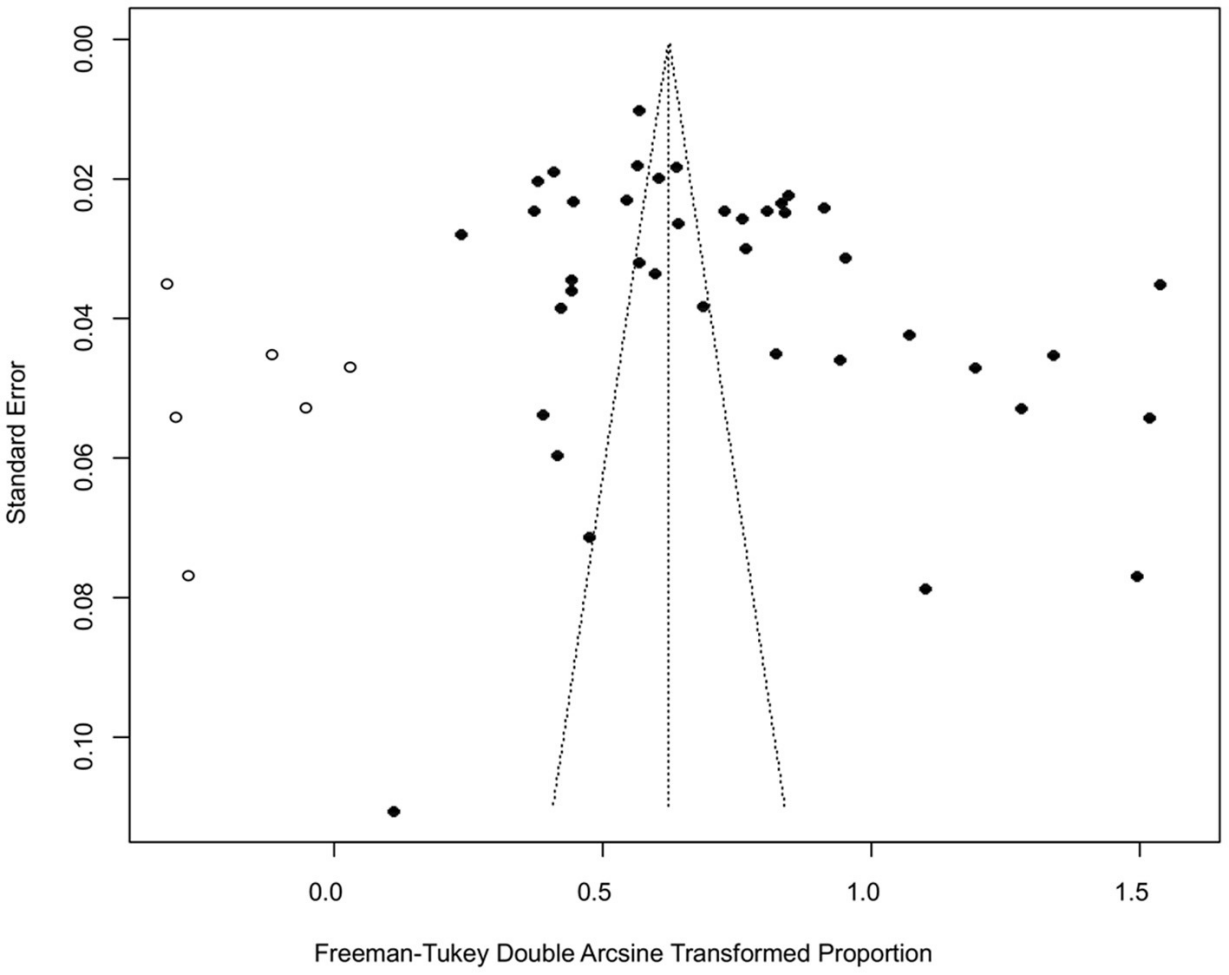
Funnel plot with trim and filling analysis of the publication bias.

**Figure 6:**
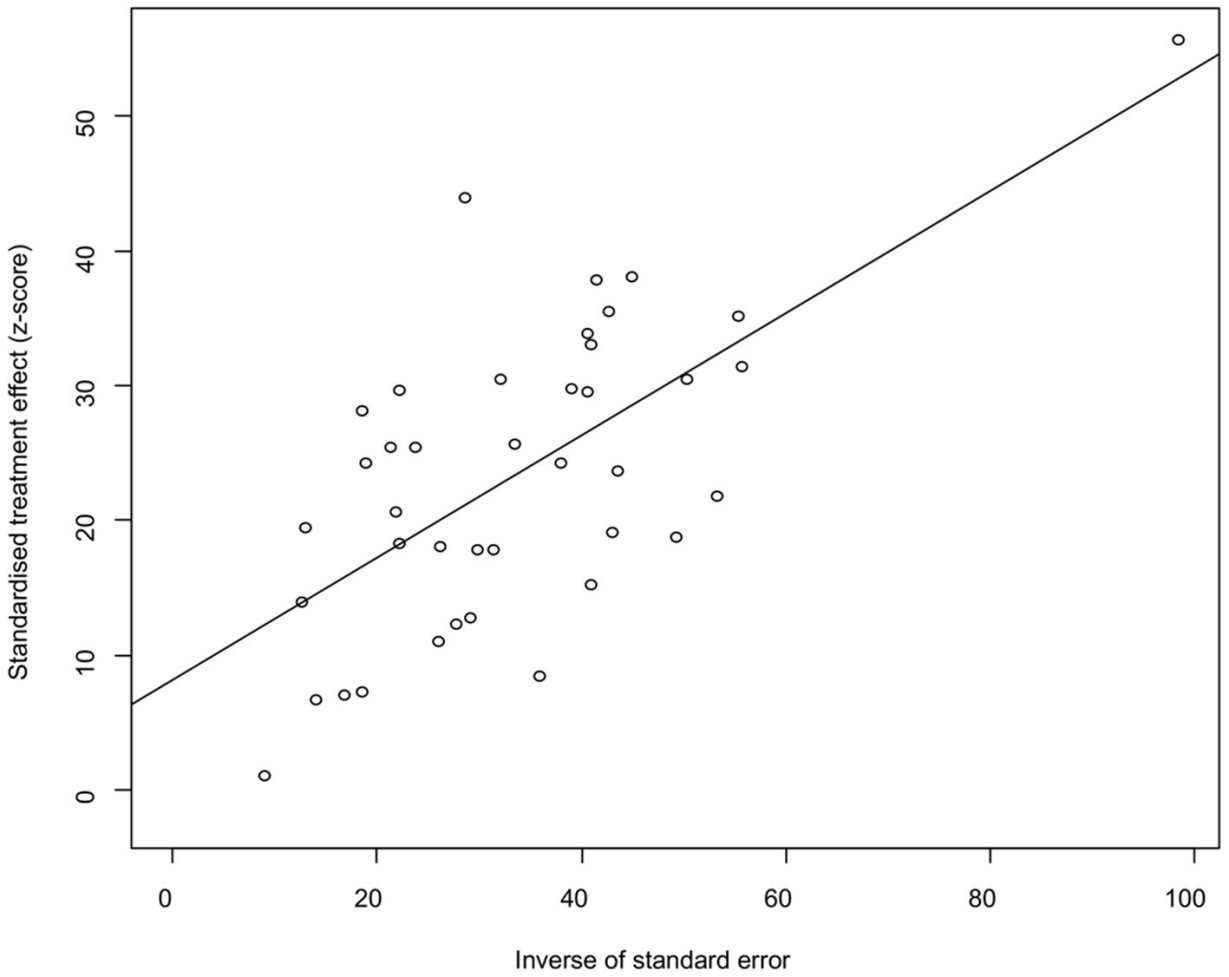
Egger's test for publication bias.

**Figure 7:**
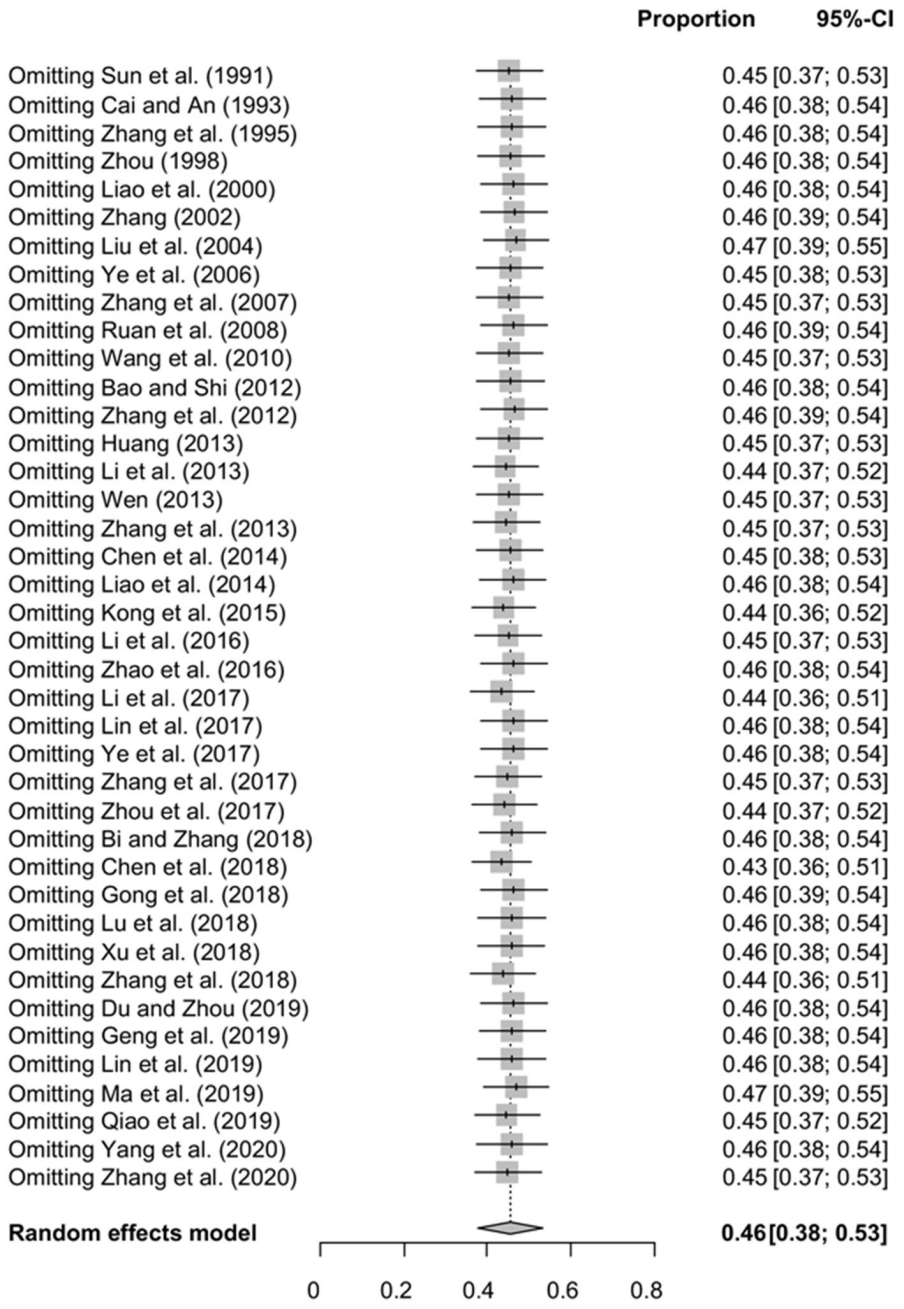
Sensitivity analysis.

The funnel plot was asymmetric, suggesting that the included studies might have publication bias or small-study effect bias (Figure 4). Meanwhile, the trim and fill analysis showed six studies with negative results (white circles in Figure 5), indicating that there was potential publication bias in the present study. Additionally, Egger's test suggested that there might be publication bias among the studies selected for our analysis (*P* < 0.05) (Supplementary Table 4, Figure 6). We also used funnel plots (Supplementary Figures 1–9) and forest plots (Supplementary Figures 10–16) for all subgroups to test for the presence of publication bias and heterogeneity. However, the sensitivity analysis showed that the pooled data were basically the same after omitting one study at a time, indicating that our results were statistically robust (Figure 7).

## Discussion

Human anisakiasis is caused by consumption of raw or poorly cooked fish parasitized by anisakid nematodes (69, 70). Hence, detailed knowledge of the epidemiological status of anisakid nematodes in fish is central for the prevention and control of human anisakiasis. Our meta-analysis revealed that the pooled estimate of Anisakidae larvae prevalence among fish in China was 45.5%, and the prevalence varied by sea areas. East China Sea and Yellow Sea had high prevalence. Fish species may contribute to such high prevalence, such as hairtail (*Trichiurus haumela*), chub mackerel (*Pneumatophorus japonicus*), yellow croaker (*Pseudosciaena polyactis*) and whitespotted conger (*Conger myriaster*) in East China Sea, and chub mackerel (*P. japonicus*) in Yellow Sea. Several previous studies showed that they were highly infected species (52, 71–73). Additionally, the relationship between the lowest prevalence in Bohai Sea and fish species needs to be further studied, because only two studies were included for analysis, and one did not disclose the 23 fish species which were tested negative for anisakid nematodes (74). A previous investigation using fish collected from three sea areas of the Republic of Korea also showed that the infection rate was higher in East Sea than that in Yellow Sea (71). However, fish from South Sea, Republic of Korea had higher prevalence rate than that from South China Sea (71). This may be due to the fact that fat greenling (*Hexagrammos otakii*) and Korean rockfish (*Sebastes schlegeli*) from South Sea with high infection rate were not included in fish species sampled from South China Sea (71). In addition to fish species, differences in prevalence may be associated with fishing grounds (15). For example, previous studies demonstrated that the distribution of *Anisakis* spp. and the infection levels in the same fish species varied among different fishing grounds (15, 75).

Among five provinces within eastern China, Zhejiang province had the highest prevalence. This may be due to the fish species, such as hairtail (*Trichiurus lepturus*) and yellow croaker (*Larimichthys polyactis*) which were reported to be highly infected species of marine fish (52). Previous studies showed that the high incidence of anisakidosis was significantly associated with living on the coast, where the habit of consuming raw fish is higher compared to inland regions (76, 77). Considering that consumption of raw or undercooked fish is a common practice in the coastal areas of China, there should be some potential cases of anisakiasis in eastern China, especially in Zhejiang province (17, 40). However, no cases of human infection by anisakid nematodes have been reported in eastern China. To date, only one case of anisakiasis has been reported in other areas of China (17). This may be due to misdiagnosis and missed diagnosis (78). Infection by anisakid nematodes should be considered in patients who had a history of ingestion of raw fish with associated symptoms, such as vomiting and frequent mucous diarrhea (17).

The method of examining fish for anisakid infection include routine visual inspection, digesting the fish filet using a pepsin/HCl solution, and incubation of internal organs (79). In all of the included studies, prevalence of anisakid nematodes in fish in China was determined by routine visual inspection. Additional species identification using PCR method was performed only in several studies. Hence, detection method as the risk factor was not included.

China released the National Agricultural and Rural Economic Development in the Tenth Five-Year Plan implemented from June 2001 (2001–2005). Of which, speeding up the development of the aquaculture industry was included. Meanwhile, establishing and perfecting a system for monitoring the safety and quality of aquatic products was mentioned. Hence, 2001 was used to be a first cut-off point for subgroup analysis. The 12th Five-Year Plan on Fishery Development and the 13th Five-Year Plan on Fishery Development were released in June 2011 and December 2016, respectively, each gives a higher priority for epidemic prevention and control of aquatic animals as well as safety and quality of aquatic products than before. Thus, we chose 2011 as the cut-off point to analyze the prevalence of anisakid nematodes. It is worth noting that we found 19 studies published after 2011, but only 5 studies before 2001. Hence, we speculated that the pooled estimates after 2011 was more likely to reflect prevalence of anisakid nematodes in fish in China.

Additionally, the rareness of anisakiasis in China may be associated with anisakid nematode species. Previous studies showed that the majority of human cases of anisakiasis were caused by *Anisakis simplex, Anisakis pegreffii*, and *Pseudoterranova decipiens* (10, 80, 81). However, *A. simplex* and *A. pegreffii* were reported only in 12 and 11 articles, respectively. The PCR approach proved to be cost-effective and reliable for the identification of the species of the genus *Anisakis* (82). However, PCR approach was not used in all studies related to species identification, which may lead to species misidentification. Moreover, only one article reported the presence of *P. decipiens* in fish in China.

Parasites were detected in muscle, intestine, mesentery and gonads. Although the point estimate of anisakid nematodes in muscle was low, larval migration to the muscles may occur after the death of the fish, which can increase the risk of anisakiasis (83, 84). Moreover, the differences between the two sibling species (*A. simplex* and *A. pegreffii*) in migration to the muscles of fish and to penetrate into the tissue of accidental hosts were found in several studies (38, 85, 86). From the perspective of food safety, further studies are needed to reveal the species composition of *Anisakis* and their geographical distribution in China.

The included studies covered a variety of fish species, and the prevalence of anisakid nematodes ranged from 0 to 100%. The results can serve as a guideline associated with food safety. Yellow goosefish (*Lophius litulon*) is a commercially important marine fish, and its stomach, intestine and liver are considered to be a delicacy in China (49). Also, cinnamon flounder (*Pseudorhombus cinnamoneus*) is a frequently consumed marine fish in China (40). Our analysis showed that *L. litulon and P. cinnamoneus* had a high prevalence, respectively. The high prevalence may be due to the fact that they eat crustaceans and small fishes, which are intermediate or paratenic hosts of anisakid nematodes (7, 11, 12). Additionally, several fish species, such as banded sergeant (*Abudefduf septemfasciatus*), sablefish (*Anoplopoma fimbria*), and skipjack tuna (*Katsuwonus pelamis*) tested negative for anisakid nematodes. This may be due to the small sample size for each of these fish species, because infection of *K. pelamis* by *Anisakis* larvae has been reported (12). Hence, further studies employing a larger number of sampled fish are needed to determine the prevalence in several fish species.

The advantages of the present study include the wide coverage, large total sample size, valid analysis method, large time span, and a comprehensive risk factor analysis. This is the first meta-analysis of the prevalence of anisakid nematodes in China. In the present study, most of the articles of medium quality reached the score of three. In addition, four or more potential risk factors were explored in the majority of articles. We believe that the study can reflect the prevalence of anisakid nematodes in fish in China during the last two decades. However, there are some limitations in this meta-analysis as follows: (i) five databases were used to identify publications, which may exclude some qualified articles from other databases; (ii) parts of the subgroups (such as sites of infection) have included fewer articles, which may lead to unstable results; (iii) this study was not registered in Cochrane, however, our meta-analysis was carried out strictly in accordance with the steps of PRISMA; and (iv) the range of environmental temperatures in the sea area where fish live is quite different from that of the land area, and analysis based on different regions of land areas may only serve as a reference. It is suggested that the researchers should clarify the sampling locations and fishing sites (such as the latitude and longitude of the specific sea area), which can contribute to the assessment of the environmental factor.

## Conclusion

This study has shown that anisakid infection in fish was widespread in China, and the pooled prevalence varied among different fish species and provinces. Region, site of infection, fish status and quality level were the main factors affecting the prevalence rate. There is a need for continuous monitoring of anisakid infection in fish in China. Meanwhile, it is necessary to educate people, especially those living in coastal regions, about the risk of infection with anisakid nematodes and to avoid consumption of raw or undercooked fish.

## Data Availability Statement

The original contributions presented in the study are included in the article/Supplementary Material, further inquiries can be directed to the corresponding authors.

## Author Contributions

Q-LG and JJ contributed to conception and design of this analysis. QL, QW, and J-YM collected the data and built the database. QW and Q-LG analyzed the results. QL prepared the manuscript. Q-LG and X-QZ revised the manuscript. All authors contributed to manuscript editing and approved the final manuscript.

## Funding

Project support was provided by the Fund for Shanxi 1331 Project (Grant No. 20211331-13), the Research Fund for Introduced High-level Leading Talents of Shanxi Province, the Special Research Fund of Shanxi Agricultural University for High-level Talents (Grant No. 2021XG001), and Yunnan Expert Workstation (Grant No. 202005AF150041).

## Conflict of Interest

The authors declare that the research was conducted in the absence of any commercial or financial relationships that could be construed as a potential conflict of interest.

## Publisher's Note

All claims expressed in this article are solely those of the authors and do not necessarily represent those of their affiliated organizations, or those of the publisher, the editors and the reviewers. Any product that may be evaluated in this article, or claim that may be made by its manufacturer, is not guaranteed or endorsed by the publisher.
