# Supplementary material for: Prevalence of Anisakid Nematodes in Fish in China: A Systematic Review and Meta-Analysis
Source: Front Vet Sci. 2022 Feb 21;9:792346. doi: 10.3389/fvets.2022.792346 (PMC8899408; doi:10.3389/fvets.2022.792346)
Supplement: Supplementary file 1 [file Data_Sheet_1.docx]

**Supplementary Table 1 |** PRISMA Checklist item.

| **Section/topic** | **#** | **Checklist item** | **Reported on page #** |
| --- | --- | --- | --- |
| **TITLE** |  |  |  |
| Title | 1 | Prevalence of anisakid nematodes in fish in China: A systematic review and meta-analysis | 1 |
| **ABSTRACT** |  |  |  |
| Structured summary | 2 | Provide a structured summary including, as applicable: background; objectives; data sources; study eligibility criteria, participants, and interventions; study appraisal and synthesis methods; results; limitations; conclusions and implications of key findings; systematic review registration number. | 2 |
| **INTRODUCTION** |  |  |  |
| Rationale | 3 | Describe the rationale for the review in the context of what is already known. | 3-4 |
| Objectives | 4 | Provide an explicit statement of questions being addressed with reference to participants, interventions, comparisons, outcomes, and study design (PICOS). | 3-4 |
| **METHODS** |  |  |  |
| Protocol and registration | 5 | Indicate if a review protocol exists, if and where it can be accessed (e.g., Web address), and, if available, provide registration information including registration number. | 4-7 |
| Eligibility criteria | 6 | Specify study characteristics (e.g., PICOS, length of follow-up) and report characteristics (e.g., years considered, language, publication status) used as criteria for eligibility, giving rationale. | 4-7 |
| Information sources | 7 | Describe all information sources (e.g., databases with dates of coverage, contact with study authors to identify additional studies) in the search and date last searched. | 4-7 |
| Search | 8 | Present full electronic search strategy for at least one database, including any limits used, such that it could be repeated. | 4-7 |
| Study selection | 9 | State the process for selecting studies (i.e., screening, eligibility, included in systematic review, and, if applicable, included in the meta-analysis). | 4-7 |
| Data collection process | 10 | Describe method of data extraction from reports (e.g., piloted forms, independently, in duplicate) and any processes for obtaining and confirming data from investigators. | 4-7, Table S2, Figure 1 |
| Data items | 11 | List and define all variables for which data were sought (e.g., PICOS, funding sources) and any assumptions and simplifications made. | 4-7 |
| Risk of bias in individual studies | 12 | Describe methods used for assessing risk of bias of individual studies (including specification of whether this was done at the study or outcome level), and how this information is to be used in any data synthesis. | 4-7 |
| Summary measures | 13 | State the principal summary measures (e.g., risk ratio, difference in means). | 4-7 |
| Synthesis of results | 14 | Describe the methods of handling data and combining results of studies, if done, including measures of consistency (e.g., I^2^) for each meta-analysis. | 6-7 |
| Risk of bias across studies | 15 | Specify any assessment of risk of bias that may affect the cumulative evidence (e.g., publication bias, selective reporting within studies). | 6-7, Figs 4-6 |
| Additional analyses | 16 | Describe methods of additional analyses (e.g., sensitivity or subgroup analyses, meta-regression), if done, indicating which were pre-specified. | 6-7 |
| **RESULTS** |  |  |  |
| Study selection | 17 | Give numbers of studies screened, assessed for eligibility, and included in the review, with reasons for exclusions at each stage, ideally with a flow diagram. | 8-10, Figure 1 |
| Study characteristics | 18 | For each study, present characteristics for which data were extracted (e.g., study size, PICOS, follow-up period) and provide the citations. | 8-10, Table S2 |
| Risk of bias within studies | 19 | Present data on risk of bias of each study and, if available, any outcome level assessment (see item 12). | 8-10, Figs 4-7, Figs S1-S9, Table S4 |
| Results of individual studies | 20 | For all outcomes considered (benefits or harms), present, for each study: (a) simple summary data for each intervention group (b) effect estimates and confidence intervals, ideally with a forest plot. | 8-10, Figure 2 |
| Synthesis of results | 21 | Present results of each meta-analysis done, including confidence intervals and measures of consistency. | 8-10, Figure 2, Tables 4 and 5 |
| Risk of bias across studies | 22 | Present results of any assessment of risk of bias across studies (see Item 15). | 8-10, Figs 4-7, Figs S1-S9, Table S4 |
| Additional analysis | 23 | Give results of additional analyses, if done (e.g., sensitivity or subgroup analyses, meta-regression [see Item 16]). | 8-10, Fig 7, Tables 4 and 5 |
| **DISCUSSION** |  |  |  |
| Summary of evidence | 24 | Summarize the main findings including the strength of evidence for each main outcome; consider their relevance to key groups (e.g., healthcare providers, users, and policy makers). | 10-14 |
| Limitations | 25 | Discuss limitations at study and outcome level (e.g., risk of bias), and at review-level (e.g., incomplete retrieval of identified research, reporting bias). | 10-14 |
| Conclusions | 26 | Provide a general interpretation of the results in the context of other evidence, and implications for future research. | 14 |
| **FUNDING** |  |  |  |
| Funding | 27 | Describe sources of funding for the systematic review and other support (e.g., supply of data); role of founders for the systematic review. | 15 |

*From:* Moher D, Liberati A, Tetzlaff J, Altman DG, The PRISMA Group (2009). Preferred Reporting Items for Systematic Reviews and Meta-Analyses: The PRISMA Statement. PLoS Med 6(6): e1000097. doi:10.1371/journal.pmed1000097

For more information, visit: **www.prisma-statement.org**.

**Supplementary Table 2 |** The code in R for this meta-analysis.

| Logarithmic conversion (PNL) | rate<-transform [m1, log=log(event/n)];  shapiro.test(rate$log) |
| --- | --- |
| Logit transformation (PLOGIT) | rate<-transform{m1, logit=log[(event/n)/(1-event/n)]};  shapiro.test(rate$logit) |
| Arcsine transformation (PAS) | rate<-transform{m1, arcsin.size=asin[sqrt(event/(n+1))]};  shapiro.test(rate$arcsin) |
| Double-arcsine transformation (PFT) | rate<-transform{m1,darcsin=0.5*[asin(sqrt(event/(n+1)))+asin((sqrt(event+1)/(n+1)))]};  shapiro.test(rate$darcsin) |
| No transformation (PRAW) | rate<-transform[m1, r= event/n];  shapiro.test(rate$r) |
| Forest plots | forest [meta1, xlim=c(-0.2, 0.8)] |
| Funnel chart | funnel (meta1) |
| Egger's test | metabias (meta1, method="linreg") |
| The sensitivity analysis | metainf (meta1, pooled = "random") forest (metainf (meta1, pooled = "random"), xlim=c(0, 0.3)) |
| Subgroup analysis | meta1<-metaprop(event, n, study, data=rate, sm="PLN", incr=0.5, allincr=TRUE, addincr=FALSE, title="", byvar= subgroup title, print.byvar=TRUE) |
| Meta-regression analysis | metareg (meta1, ~covariate title) |

**Supplementary Table 3 |** Included studies of anisakid infection in fish in China.

| **Study No.** | **Study ID** | **Quality score** | **No. examined** | **No. positive** | **Prevalence** | **Sampled randomly or not** | **Detection method clearly or not** | **Sampled method detailedly or not** | **Sampled time clearly or not** | **Four or more risk factors or not** | **Quality score** | **Study design** |
| --- | --- | --- | --- | --- | --- | --- | --- | --- | --- | --- | --- | --- |
| 1 | Bao and Shi (2012) | High | 413 | 182 | 0.4407 | N* | Y* | Y | N | Y | 5 | Cross sectional |
| 2 | Bi and Zhang (2018) | High | 246 | 71 | 0.2886 | Y | N | Y | Y | Y | 4 | Cross sectional |
| 3 | Cai and An (1993) | High | 474 | 126 | 0.2658 | N | Y | Y | Y | Y | 4 | Cross sectional |
| 4 | Chen et al. (2014) | High | 382 | 181 | 0.4738 | Y | Y | Y | Y | Y | 5 | Cross sectional |
| 5 | Du and Zhou (2019) | High | 193 | 35 | 0.1813 | Y | Y | Y | Y | Y | 5 | Cross sectional |
| 6 | Geng et al. (2019) | High | 222 | 70 | 0.3153 | N | Y | Y | Y | Y | 4 | Cross sectional |
| 7 | Gong et al. (2018) | High | 708 | 112 | 0.1582 | N | Y | Y | Y | Y | 4 | Cross sectional |
| 8 | Huang (2013) | High | 410 | 226 | 0.5512 | Y | Y | Y | Y | Y | 5 | Cross sectional |
| 9 | Li et al. (2013) | High | 113 | 98 | 0.8673 | N | Y | Y | Y | Y | 4 | Cross sectional |
| 10 | Li et al. (2016) | High | 430 | 269 | 0.6256 | Y | Y | Y | Y | Y | 5 | Cross sectional |
| 11 | Liao et al. (2014) | High | 49 | 10 | 0.2041 | N | Y | Y | Y | Y | 4 | Cross sectional |
| 12 | Lin et al. (2017) | High | 463 | 85 | 0.1836 | N | Y | Y | Y | Y | 4 | Cross sectional |
| 13 | Lin et al. (2019) | High | 763 | 269 | 0.3526 | Y | Y | Y | Y | Y | 5 | Cross sectional |
| 14 | Lu et al. (2018) | High | 633 | 204 | 0.3223 | Y | Y | Y | Y | Y | 5 | Cross sectional |
| 15 | Ruan et al. (2008) | High | 86 | 12 | 0.1395 | Y | Y | Y | Y | Y | 5 | Cross sectional |
| 16 | Wang et al. (2010) | High | 420 | 218 | 0.5190 | Y | Y | Y | Y | Y | 5 | Cross sectional |
| 17 | Wen (2013) | High | 506 | 283 | 0.5593 | N | Y | Y | Y | Y | 4 | Cross sectional |
| 18 | Xu et al. (2018) | High | 360 | 128 | 0.3556 | N | Y | Y | Y | Y | 4 | Cross sectional |
| 19 | Yang et al. (2020) | High | 2422 | 695 | 0.2870 | Y | Y | N | Y | Y | 4 | Cross sectional |
| 20 | Ye et al. (2006) | High | 281 | 135 | 0.4804 | Y | Y | N | Y | Y | 4 | Cross sectional |
| 21 | Ye et al. (2017) | High | 169 | 28 | 0.1657 | N | Y | Y | Y | Y | 4 | Cross sectional |
| 22 | Zhang et al. (2012) | High | 418 | 55 | 0.1316 | Y | Y | Y | Y | Y | 5 | Cross sectional |
| 23 | Zhang et al. (2017) | High | 256 | 170 | 0.6641 | Y | Y | Y | Y | Y | 5 | Cross sectional |
| 24 | Zhao et al. (2016) | High | 211 | 38 | 0.1801 | Y | Y | N | Y | Y | 4 | Cross sectional |
| 25 | Zhou (1998) | High | 172 | 69 | 0.4012 | N | Y | Y | Y | Y | 4 | Cross sectional |
| 26 | Zhou et al. (2017) | High | 89 | 82 | 0.9213 | N | Y | Y | Y | Y | 4 | Cross sectional |
| 27 | Chen et al. (2018) | Middle | 204 | 204 | 1.0000 | N | Y | Y | N | Y | 3 | Cross sectional |
| 28 | Kong et al. (2015) | Middle | 122 | 116 | 0.9508 | N | Y | N | Y | Y | 3 | Cross sectional |
| 29 | Li et al. (2017) | Middle | 85 | 85 | 1.0000 | N | Y | N | Y | Y | 3 | Cross sectional |
| 30 | Liao et al. (2000) | Middle | 70 | 11 | 0.1571 | N | Y | N | Y | Y | 3 | Cross sectional |
| 31 | Liu et al. (2004) | Middle | 322 | 17 | 0.0528 | N | Y | N | N | Y | 2 | Cross sectional |
| 32 | Ma et al. (2019) | Middle | 20 | 0 | 0.0000 | Y | N | N | Y | Y | 3 | Cross sectional |
| 33 | Qiao et al. (2019) | Middle | 140 | 108 | 0.7714 | N | Y | N | N | Y | 2 | Cross sectional |
| 34 | Sun et al. (1991) | Middle | 137 | 41 | 0.2993 | N | Y | N | Y | Y | 3 | Cross sectional |
| 35 | Zhang (2002) | Middle | 176 | 83 | 0.4716 | N | Y | N | Y | Y | 3 | Cross sectional |
| 36 | Zhang et al. (1995) | Middle | 777 | 221 | 0.2844 | N | Y | N | N | Y | 2 | Cross sectional |
| 37 | Zhang et al. (2007) | Middle | 123 | 66 | 0.5366 | N | Y | N | Y | Y | 3 | Cross sectional |
| 38 | Zhang et al. (2013) | Middle | 40 | 32 | 0.8000 | N | Y | N | Y | Y | 3 | Cross sectional |
| 39 | Zhang et al. (2018) | Middle | 42 | 42 | 1.0000 | N | Y | N | N | Y | 2 | Cross sectional |
| 40 | Zhang et al. (2020) | Middle | 119 | 78 | 0.6555 | N | Y | N | Y | Y | 3 | Cross sectional |

**Supplementary Table 4 |** Egger’s for publication bias.

| slope | bias | se. bias | t | df | P-value |
| --- | --- | --- | --- | --- | --- |
| 0.454 | 8.094 | 3.142 | 2.576 | 38 | 0.014 |

1. Bao, M., and Shi, K.S. (2012). Investigation on Anisakis nematode infections in sea fishes sold in Jinzhou City. Chinese Journal of Zoonoses, 28(05), 513-514+516. (In Chinese)
2. Bi, H.J., and Zhang, Y.M. (2018). Results of risk monitoring of food microorganisms and their pathogenic factors in Cangzhou City in 2017. Occupation and Health, 34(14), 1917-1920. (In Chinese)
3. Cai, Z.X., and An, S.R. (1993). Investigation on the transmission vector of Anisakis disease. Chinese Journal of Public Health, 11(05), 284-285. (In Chinese)
4. Chen, J.H., Xu, Z.X., Xu, G.X., Huang, J.Y., Chen, H.H., Shi, S.Z., et al. (2014). Survey of simple anisakis larvae infection in marine fish in Shantou. Chinese Journal of Parasitology and Parasitic Diseases, 32(03), 212-216. (In Chinese)
5. Du, X.T., and Zhou, Q.Y. (2019). Infection of the third stage larvae of heteronema in some sea fishes in Dandong City. China Tropical Medicine, 19(01), 40-42. (In Chinese)
6. Geng, Y.Z., Li, F., Wang, W.J., and Zhang, M.M. (2019). Investigation and molecular identification of Anisakis infection in marine fish in Liaoning Province. Chinese Journal of Food Hygiene, 31(01), 10-13. (In Chinese)
7. Gong, C.B., Wang, Z.X., Dong, F.G., Xing, Y.F., and Sun, Y.L. (2018).Infection status of third stage larvae of heteronema in 140 fresh marine fish sold in Yantai from 2016 to 2017. Modern Preventive Medicine, 45(10), 1766-1768. (In Chinese)
8. Huang, G.P. (2013). Molecular identification and genetic relationship analysis of the larvae of parasitic heterophyllus nematode in the order perciformes in the southern China sea. Hebei Normal University. (In Chinese)
9. Li, J., Guo, J.N., Zhou, J.B., Shi, W., Li, W.W., Fang, F., et al. (2013). A preliminary investigation on the third stage larvae of heteromynchus japonicus infected with mackerel in the Yellow Sea. Chinese Journal of Food Hygiene, 25(01), 56-61. (In Chinese)
10. Li, X.J., Shen, Y.Y., Bai, J., Chen, L.M., Zhou, Y., Shen, C.L., et al. (2016). Investigation of Anisakis larvae infection in marine fish entering and leaving the Zhoushan Port. Animal Husbandry and Veterinary Medicine, 48(05), 119-122. (In Chinese)
11. Liao, F., Zhang, B.G., Liu, X., Chen, X.X., and Fei, Y.K. (2014). Investigation on the infection of Isoapex nematode in aquatic products in yellow Sea area of Shandong Province. Parasitoses and Infectious Diseases, 12(04), 189-190. (In Chinese)
12. Lin, C.X., Lin, S.H., Chen, W.W., Huang, S.L., and Jiang, D.W. (2017). Parasite pollution in aquatic products marketed in Fujian Province, Chinese Journal of Zoonoses, 33(06), 564-568. (In Chinese)
13. Lin, C.X., Huang, S.L., Lin, S.H., Jiang, D.W., and Xie, H.G. (2019). Investigation on the infection of anisoderma larvae and identification of the species in Fujian coastal fishes, Chinese Journal of Parasitology and Parasitic Diseases, 37(04), 417-421. (In Chinese)
14. Lu, L., Jiang, S.F., He, Y.Y., Zhang, X.P., Ma, X.J., Han, Y.J., et al. (2018). Investigation on parasite infection of animal food sold in Huangpu district, Shanghai from 2015 to 2017. Journal of Tropical Diseases and Parasitology, 16(01), 23-25. (In Chinese)
15. Ruan, Y.Q., Zhang, H.M., Tan, Y.G., Huang, F.M., Lin, R., Ou, Y.Y., et al. (2008). Preliminary investigation on the infection of Marine fish with echinodiasis in Guangxi. Applied Preventive Medicine, 14(03), 147-148. (In Chinese)
16. Wang, J.Y., Zhang, J.H., Lin, Q., Zhang, Q.T., He, W.X., Li, K.F., et al. (2010). Infection and physico—chemical characteristics of Anisakis among marine fish caught in Zhoushan Fisher. Chinese Journal of Epidemiology, 30(09), 1001-1004. (In Chinese)
17. Wen, Q. (2013). Morphological and Molecular Characterization of Anisakidae Larvae from in Taiwan Strait and the Analysis of infection Status. Hebei Normal University. (In Chinese)
18. Xu, Y., Zeng, Q.Y., Sun, Z.H., Zhang, L.X., Wang, N., Liu, X.L., et al. (2018). Preliminary investigation on the infection of Anemone elegans in sea fish in Lianyungang. Chinese Journal of Veterinary Science, 38(12), 2343-2347. (In Chinese)
19. Yang, S.R., Pei, X.Y., Li, Y., Li., Z., Tang, Z., Chen, W.W., et al. (2020). Epidemical study of third stage larvae of Anisakis spp. infection in marine fishes in China from 2016 to 2017. Food Control, 107.
20. Ye, L.P., Sun, F., Xu, G.Z., Sun, Y.W., Chen, Z.H., Lu, F., et al. (2006). Investigation on Anisakis Infection of East China Sea Fish and Study on the Tolerance of Larvae to Wasabi. China Tropical Medicine, 6(08), 1345-1346 + 1512. (In Chinese)
21. Ye, B., Sun, Z.Q., Song, X.Y., Shi, X.X., Li, D.D., Xiao, N., et al. (2017). Investigation of Anisakis Larvae Infection in Commercial Marine Fish in Qingdao City, Shandong. Journal of Medical Pest Control, 33(09), 985-986. (In Chinese)
22. Zhang, X.P., Jiang, S.F., Hong, G.B., Fu, Y.H., He, Y.Y., Ma, X.J., et al. (2012). Investigation on food contamination with parasites in Shanghai market. Chinese Journal of Schistosomiasis Control, 24(04), 404-409. (In Chinese)
23. Zhang, Q.W., Ren, X.T., Zhao, Y.Q., Gai, X.X., Zhang, Y.M., and Bai, X.L. (2017). Investigation of Anisakis spp. larva infection in marine fish for sale in Yantai City. Chinese Journal of Parasitology and Parasitic Diseases, 35(05), 472-477. (In Chinese)
24. Zhao, W.T., Lv, L., Chen, H.X., Yang, Y., Zhang, L.P., and Li, L. (2016). Ascaridoid parasites infecting in the frequently consumed marine fishes in the coastal area of China: A preliminary investigation. Parasitology International, 65(2), 87-98.
25. Zhou, X.F. (1998). Investigation of Anisakis Larvae Infection in Marine Fish in Ningbo Market. Parasitoses and Infectious Diseases, 6(02), 54. (In Chinese)
26. Zhou, J.Y., Lin, Q., Zhang, H., Zhang, J.H., and Gu, Z.X. (2017). Investigation and molecular identification of anisakis infection in marine fishes in Zhoushan fishing ground. Preventive Medicine, 29(07), 694-697+701. (In Chinese)
27. Chen, H.X., Zhang, L.P., Gibson, D.I., Lv, L., Xu, Z., Li, H.T., et al. (2018). Detection of ascaridoid nematode parasitesin the important marine food-fish Congermyriaster (Brevoort) (Anguilliformes:Congridae) from the Zhoushan Fishery,China. Parasit Vectors, 11(01), 274.
28. Kong, Q.M., Fan, L.F., Zhang, J., Akao, N., Dong, K., Lou, D., et al. (2015). Molecular identification of Anisakis and Hysterothylacium larvae in marinefishes from the East China Sea and the Pacific coast of central Japan. International Journal of Food Microbiology, 199, 1-7.
29. Li, L., Zhao, J.Y., Chen, H.Y., Ju, H.D., An, M., Xu, Z., et al. (2017). Survey for the presence of ascaridoid larvae in the cinnamon flounder Pseudorhombus cinnamoneus (Temminck and Schlegel) (Pleuronectiformes: Paralichthyidae). International Journal of Food Microbiology, 241, 108-116.
30. Liao, Y.M., Li, D.L., and Zhang, X.L. (2000). An investigation on the infection of the larvae of heteromynchus spp. in the coastal waters of Nan'ao Town, Shenzhen. South China Journal of Preventive Medicine, 26(04), 46-47. (In Chinese)
31. Liu, J.S., Wu, S.Q., Chen, H.H., Lin, R.Q., Wei, D.X., Deng, Y., et al. (2005). Investigation of Anisakis larvae infection in marine fish in Daya Bay. China Anim Livest Vet Med, 24(07), 39. (In Chinese)
32. Ma, X.M., Wang, J.J., Xiao, G.Y., Zhao, J., and Li, J. (2019). Analysis of monitoring results of pathogenic microorganisms of animal aquatic products in fengtai district, Beijing. Chinese Journal of Health Laboratory Technology, 29(22), 2798-2801. (In Chinese)
33. Qiao, Y., Zhou, Q.J., Li, X.J., Miao, L., and Cheng, T. (2019). The establishment of a method for detecting simple Anisakis/Anisakis spp. with loop-mediated isothermal amplification and lateral flow test strips. Oceanologia et Limnologia Sinica, 50(02), 324-335. (In Chinese)
34. Sun, S.Z., Koyama, T., and Kagei, N. (1991). Anisakidae larvae found in marine fishes and squids from the Gulf of Tongking, the East China Sea and the Yellow Sea. Japanese Journal of Medical Science and Biology, 44(3), 99.
35. Zhang, L. (2002). Preliminary investigation on the infection of simple anisakis larvae in Bohai fish. Journal of Cangzhou Normal University, 18(03), 41-47. (In Chinese)
36. Zhang, B.X., Ge, L.M., Chen, F.Y., Sun, Y.H., Zhang, M., Yu, Y.L., et al. (1995). A preliminary study on the ecological distribution of anisakis in marine fish. Chinese Journal of Microecology, 7(04), 53-55. (In Chinese)
37. Zhang, L.P., Hu, M., Shamsi, S., Beveridge, I., Li, H.M., Xu, Z., et al. (2007). The specific identification of anisakid larvae from fishes from the Yellow Sea, China, using mutation scanning-coupled sequence analysis of nuclear ribosomal DNA. Molecules and Cellular Probes, 21(5-6), 386-390.
38. Zhang, Z.J., Zhang, W.B., Zhao, R.M., Shen, M.X., Jiang, W.C., Jin, F., et al. (2013). Status of Clonorchis sinensis and Nematoda in common fishes in Nantong City. Chinese Journal of Preventive Medicine, 47(07), 669. (In Chinese)
39. Zhang, K., Xu, Z., Chen, H.X., Guo, N., and Li, L. (2018). Anisakid and raphidascaridid nematodes (Ascaridoidea) infection in the important marine food-fish Lophius litulon (Jordan) (Lophiiformes: Lophiidae). International Journal of Food Microbiology, 284, 105-111.
40. Zhang, X.Y., Yu, M., Zhao, Q.Q., Wang, Y., and Sun, B.C. (2020). Investigation of Anisakis Infection in Marine Fishes in Dongtai City. Chinese Journal of Schistosomiasis Control, 32(04), 426-427,440. (In Chinese)
