# Supplementary material for: Prevalence of Anisakid Nematodes in Fish in China: A Systematic Review and Meta-Analysis
Source: Front Vet Sci. 2022 Feb 21;9:792346. doi: 10.3389/fvets.2022.792346 (PMC8899408; doi:10.3389/fvets.2022.792346)
Supplement: Supplementary file 2 [file Data_Sheet_2.docx]

0.5

1.0

1.5

0.10

0.08

0.06

0.04

0.02

0.00

Freeman-Tukey Double Arcsine Transformed Proportion

Standard Error

**Supplementary Figure 1 |** Funnel plot with pseudo 95% confidence limits intervals for the examination of publication bias of region.

0.5

1.0

1.5

0.10

0.08

0.06

0.04

0.02

0.00

Freeman-Tukey Double Arcsine Transformed Proportion

Standard Error

**Supplementary Figure 2 |** Funnel plot with pseudo 95% confidence limits intervals for the examination of publication bias of sampling years.

0.0

0.2

0.4

0.6

0.8

1.0

1.2

0.07

0.06

0.05

0.04

0.03

0.02

0.01

0.00

Freeman-Tukey Double Arcsine Transformed Proportion

Standard Error

**Supplementary Figure 3 |** Funnel plot with pseudo 95% confidence limits intervals for the examination of publication bias of site of infection.

0.4

0.6

0.8

1.0

1.2

1.4

0.12

0.10

0.08

0.06

0.04

0.02

0.00

Freeman-Tukey Double Arcsine Transformed Proportion

Standard Error

**Supplementary Figure 4|** Funnel plot with pseudo 95% confidence limits intervals for the examination of publication bias of season.

0.4

0.6

0.8

1.0

1.2

1.4

1.6

0.06

0.04

0.02

0.00

Freeman-Tukey Double Arcsine Transformed Proportion

Standard Error

**Supplementary Figure 5 |** Funnel plot with pseudo 95% confidence limits intervals for the examination of publication bias of sea.

0.5

1.0

1.5

0.10

0.08

0.06

0.04

0.02

0.00

Freeman-Tukey Double Arcsine Transformed Proportion

Standard Error

**Supplementary Figure 6 |** Funnel plot with pseudo 95% confidence limits intervals for the examination of publication bias of fish status.

0.5

1.0

1.5

0.10

0.08

0.06

0.04

0.02

0.00

Freeman-Tukey Double Arcsine Transformed Proportion

Standard Error

**Supplementary Figure 7 |** Funnel plot with pseudo 95% confidence limits intervals for the examination of publication bias of quality level.

0.5

1.0

1.5

0.10

0.08

0.06

0.04

0.02

0.00

Freeman-Tukey Double Arcsine Transformed Proportion

Standard Error

**Supplementary Figure 8 |** Funnel plot with pseudo 95% confidence limits intervals for the examination of publication bias of sampling provinces.

0.0

0.5

1.0

1.5

0.4

0.3

0.2

0.1

0.0

Freeman-Tukey Double Arcsine Transformed Proportion

Standard Error

**Supplementary Figure 9 |** Funnel plot with pseudo 95% confidence limits intervals for the examination of publication bias of species of fish.

**
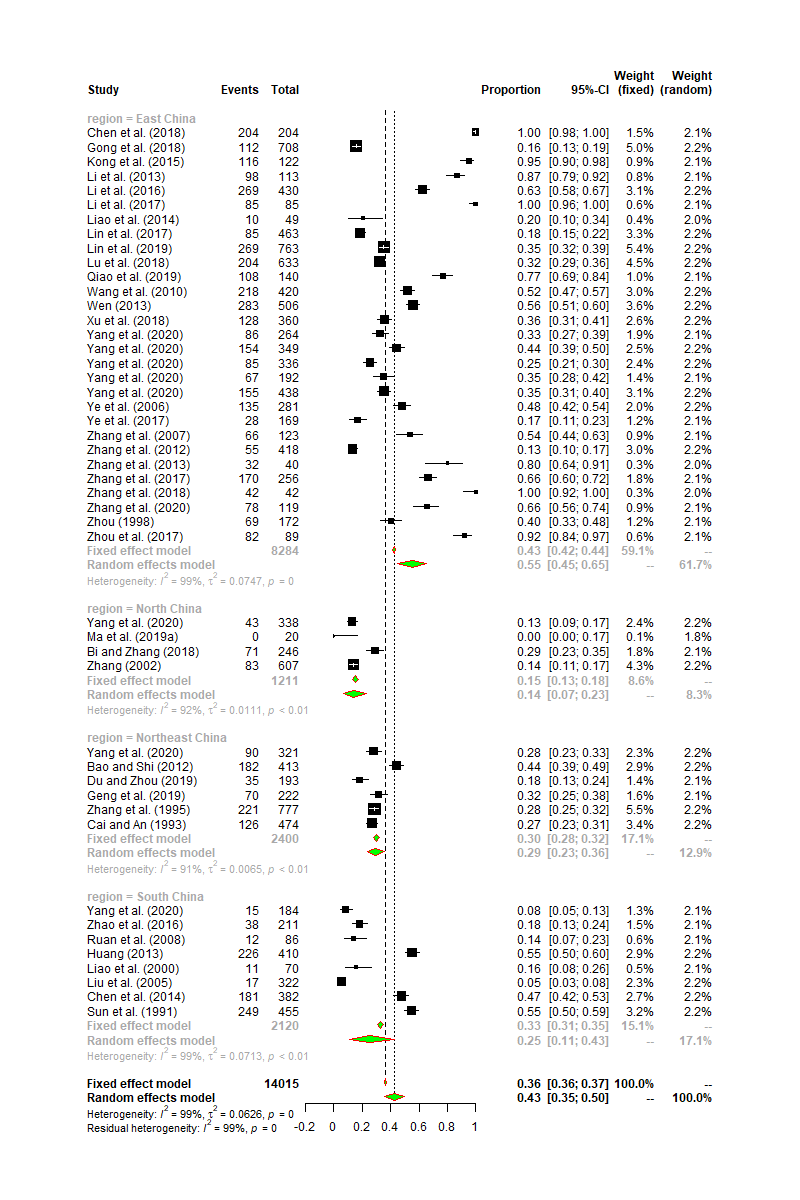
**

**Supplementary Figure 10 |** Forest plot of prevalence of anisakid nematodes in fish for region.


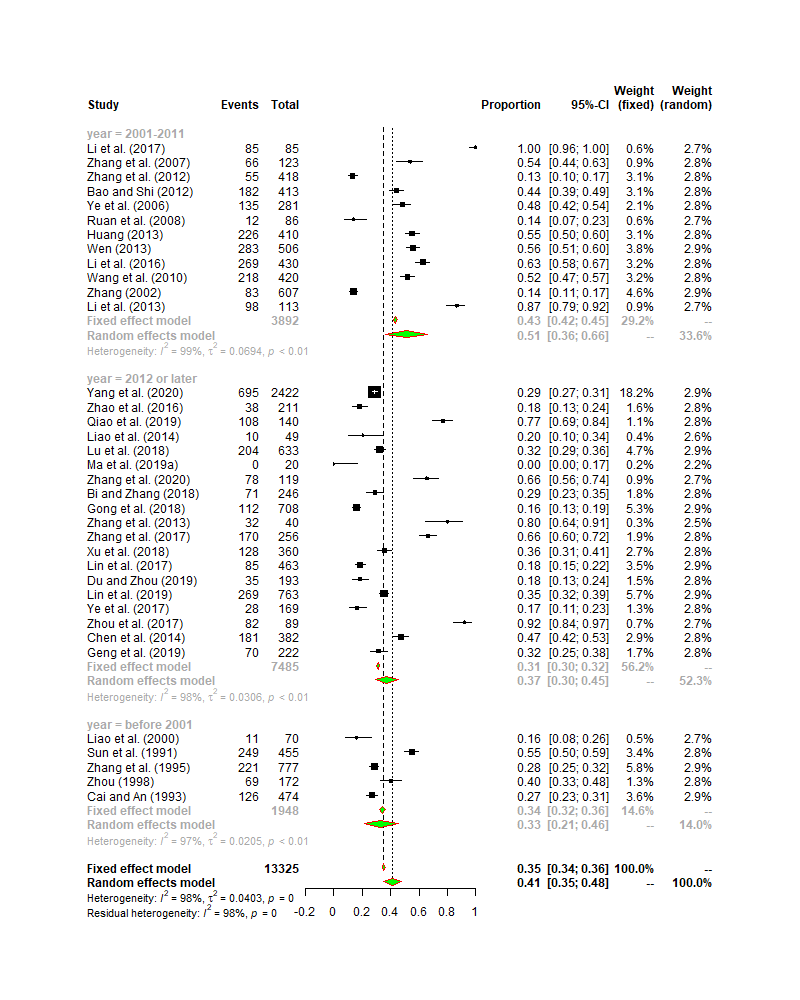


**Supplementary Figure 11 |** Forest plot of prevalence of anisakid nematodes in fish for sampling years.

**
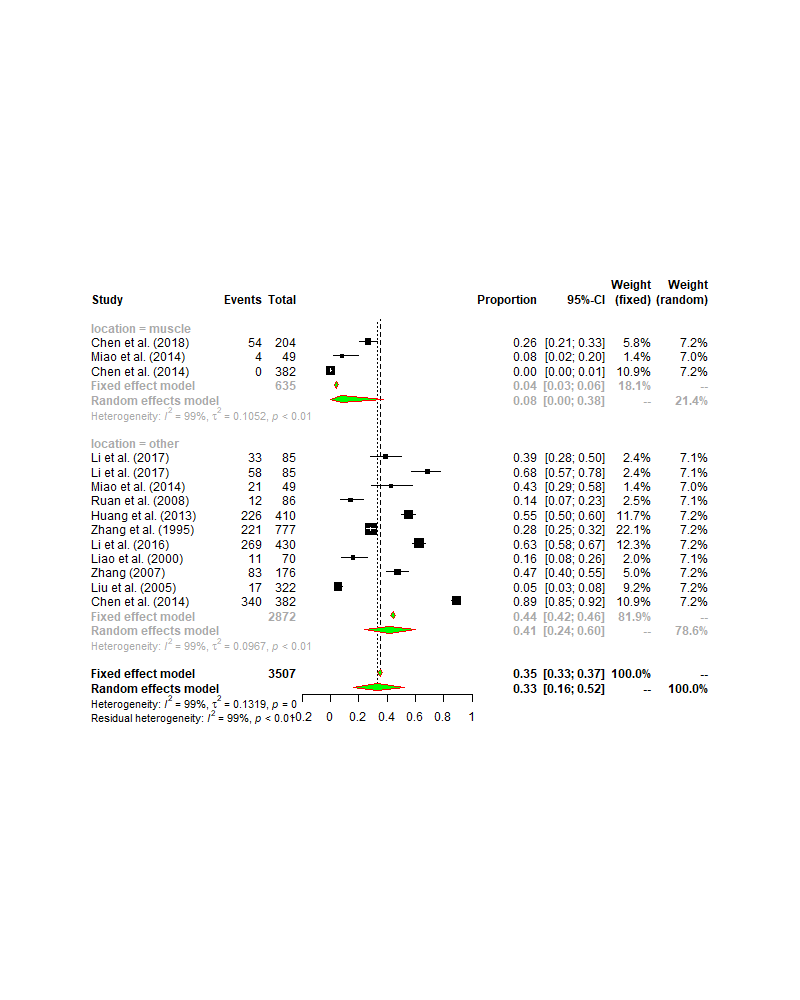
**

**Supplementary Figure 12 |** Forest plot of prevalence of anisakid nematodes in fish for site of infection.


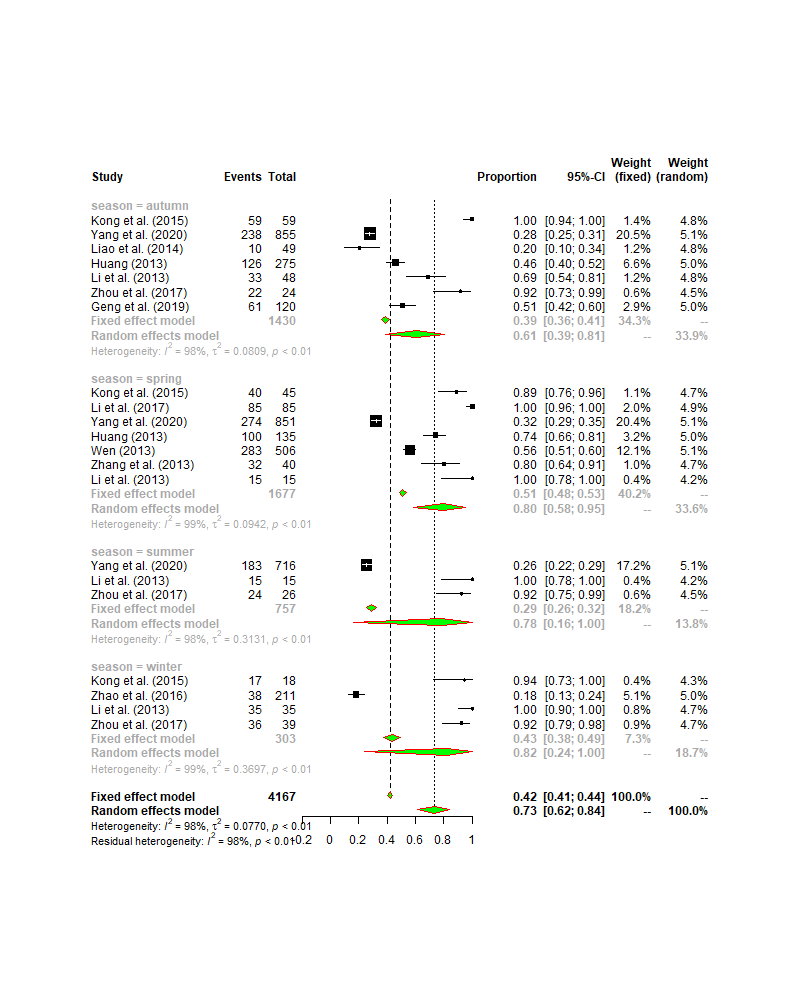


**Supplementary Figure 13 |** Forest plot of prevalence of anisakid nematodes in fish for season.

**
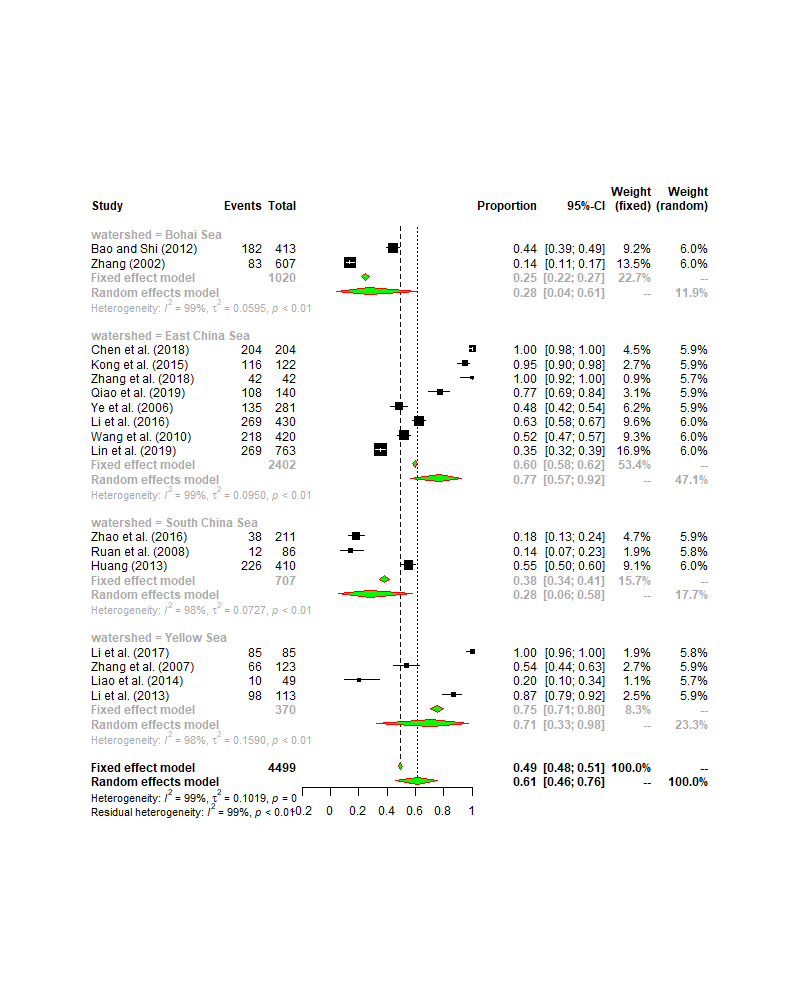
**

**Supplementary Figure 14 |** Forest plot of prevalence of anisakid nematodes in fish for sea.


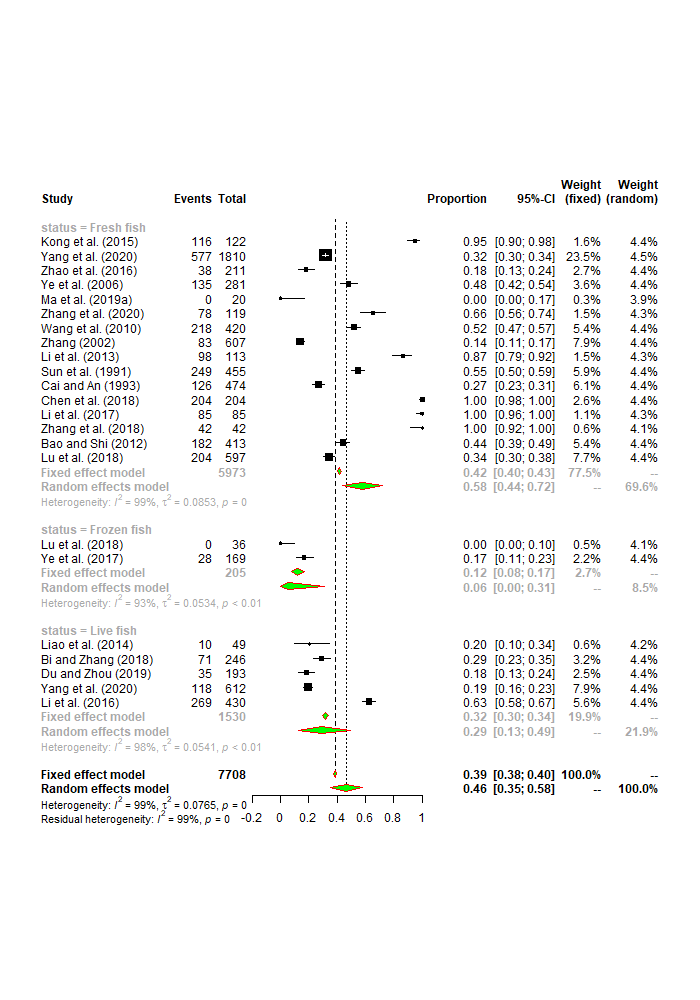


**Supplementary Figure 15 |** Forest plot of prevalence of anisakid nematodes in fish for fish status.


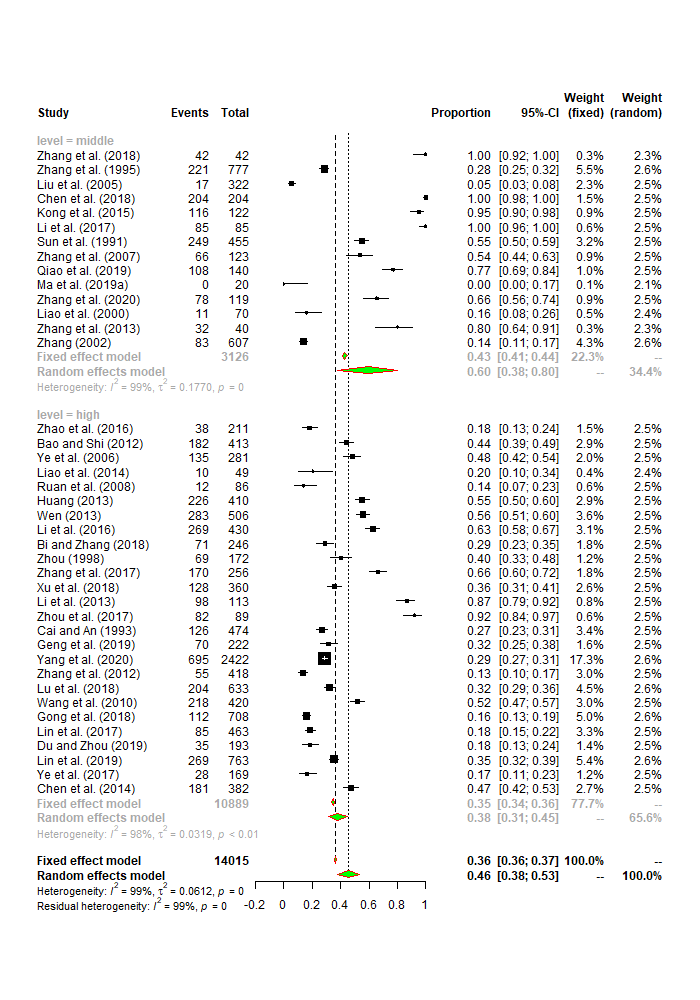


**Supplementary Figure 16 |** Forest plot of genera of anisakid nematodes in fish for quality level.
